# Preclinical Evaluation of Novel Folate Receptor 1-Directed CAR T Cells for Ovarian Cancer

**DOI:** 10.3390/cancers16020333

**Published:** 2024-01-12

**Authors:** Julie Daigre, Manuel Martinez-Osuna, Maria Bethke, Larissa Steiner, Vera Dittmer, Katrin Krischer, Cathrin Bleilevens, Janina Brauner, Jens Kopatz, Matthias David Grundmann, Paurush Praveen, Dominik Eckardt, Andreas Bosio, Christoph Herbel

**Affiliations:** Miltenyi Biotec B.V. & Co. KG, Friedrich-Ebert-Strasse 68, 51429 Bergisch Gladbach, Germany; julieda@miltenyi.com (J.D.); manuelma@miltenyi.com (M.M.-O.); mariabet@miltenyi.com (M.B.); larissas@miltenyi.com (L.S.); verac@miltenyi.com (V.D.); katrinsch@miltenyi.com (K.K.); cathrinl@miltenyi.com (C.B.); janinak@miltenyi.com (J.B.); jensko@miltenyi.com (J.K.); matthiasgr@miltenyi.com (M.D.G.); paurushp@miltenyi.com (P.P.); dominike@miltenyi.com (D.E.); andreasbo@miltenyi.com (A.B.)

**Keywords:** ovarian cancer, immunotherapy, cancer cell therapy, CAR T cells, FOLR1, cell surface targets, solid tumor

## Abstract

**Simple Summary:**

Ovarian cancer is a devastating disease due to the late diagnosis of advanced stage disease and high recurrence rates. Thus, new long-lasting, efficient drugs are needed. Since chimeric antigen receptor (CAR)-expressing T cell treatments have led to efficient and persisting anti-tumor responses, our study aimed to design and characterize CAR T cells targeting ovarian cancer. We confirmed folate receptor 1 (FOLR1) as a promising tumor-associated antigen in ovarian cancer patient samples. After designing a library of FOLR1-directed CAR T cells, their functionality and specificity were assessed in vitro against cell lines as well as in vivo in a mouse model of ovarian cancer. Finally, we selected a lead candidate for further characterization. The anti-FOLR1 CAR was successfully tested, including assays, reflecting the solid tumor microenvironment. These characteristics will support the clinical translation of our new FOLR1-specific CAR T cell asset.

**Abstract:**

Treatment options for ovarian cancer patients are limited, and a high unmet clinical need remains for targeted and long-lasting, efficient drugs. Genetically modified T cells expressing chimeric antigen receptors (CAR), are promising new drugs that can be directed towards a defined target and have shown efficient, as well as persisting, anti-tumor responses in many patients. We sought to develop novel CAR T cells targeting ovarian cancer and to assess these candidates preclinically. First, we identified potential CAR targets on ovarian cancer samples. We confirmed high and consistent expressions of the tumor-associated antigen FOLR1 on primary ovarian cancer samples. Subsequently, we designed a series of CAR T cell candidates against the identified target and demonstrated their functionality against ovarian cancer cell lines in vitro and in an in vivo xenograft model. Finally, we performed additional in vitro assays recapitulating immune suppressive mechanisms present in solid tumors and developed a process for the automated manufacturing of our CAR T cell candidate. These findings demonstrate the feasibility of anti-FOLR1 CAR T cells for ovarian cancer and potentially other FOLR1-expressing tumors.

## 1. Introduction

Epithelial ovarian cancer (OvCa) is the most deadly gynecological cancer and the fifth-leading cause of cancer deaths in women [1]. A high unmet medical need exists due to the late diagnosis of its advanced-stage disease (stage III-IV), and high relapse rates after first-line treatment, which ultimately results in 5-year survival rates of 46% [1]. The most frequent histological subtype of epithelial OvCa is high-grade serous OvCa, which accounts for 60–80% of all cases [2].

Current treatment options are still limited, and only recently, targeted therapies, namely vascular endothelial growth factor (VEGF)/vascular endothelial growth factor receptor (VEGFR) pathway inhibitors and poly (ADP-ribose) polymerase (PARP) inhibitors, have been approved and become available. These targeted therapeutic approaches have demonstrated clinically meaningful improvements and, at the same time, have presented with improved safety profile over systemic chemotherapy [3]. Thus, it is important to stratify patients to better direct treatment strategies. Proper target selection is critical for anti-tumor efficacy and safety, sparing non-malignant cells.

Previous studies have shown that Folate receptor 1 (FOLR1, also FRα), which is a glycophosphatidylinositol (GPI)-linked membrane glycoprotein mediating the uptake of reduced folates and folic acid [4], is an attractive antigen for targeted therapeutic approaches since FOLR1 is aberrantly expressed in several epithelial tumors, including OvCa [5]. In normal, healthy tissues, the expression of FOLR1 is limited and restricted to the luminal side of a subpopulation of epithelial cells, primarily in kidney and lung tissues. There, FOLR1 is regarded as not accessible to FOLR1-targeted agents in circulation [6]. Therefore, FOLR1 is considered a safe target due to its low or absent expression in non-malignant cells [7], and FOLR1 expression is not altered after chemotherapy [8], which is the standard-of-care treatment for OvCa [9]. Immunotherapeutic strategies targeting FOLR1 demonstrated clinical efficacy with minimal toxicities [10].

Consequently, the first therapeutic monoclonal antibody targeting FOLR1, MORAb-003, was developed and safely applied in patients [11]. However, this approach lacked anti-tumor efficacy [11]. More recently, a new class of therapeutic molecules, antibody-drug conjugates (ADCs), with increased potency is under clinical investigation. ADCs are capable of delivering a cytotoxic agent in an antigen-dependent manner. One example of a FOLR1-targeting ADC for OvCa is IMGN853, also known as Mirvetuximab soravtansine or Elahere^TM^, which received FDA approval in 2022 [12,13]. However, this approach requires high target antigen expressions, the cytotoxic payload acts solely on dividing cells, and its anti-tumor function may not persist after the discontinuation of the treatment.

Alternatively, cellular immunotherapy is a promising targeted approach to increase persistence and durable responses in patients. The genetic modification of T cells to express chimeric antigen receptors (CARs) has shown efficacious, as well as persisting, anti-tumor function in many patients suffering from hematological cancers [14]. The first studies used genetically modified T cells to target FOLR1 [15]. This approach was safe in patients but lacked anti-tumor efficacy. The lack of efficacy was attributed to poor persistence accompanied by human anti-mouse antibodies present in the patient [16]. Nevertheless, our understanding of CAR T cell design and manufacturing has advanced, and next-generation CAR T cells have optimized functionality and persistence [17,18].

Here, we aim to overcome the limitations of previous CAR T cell approaches targeting FOLR1, namely poor persistence and lack of efficacy by employing human and humanized CAR sequences instead of murine sequences to prevent a potential human anti-mouse immune response. Moreover, we employed innovative CAR design strategies utilizing human promoter sequences in contrast to viral promoter sequences to potentially enhance the efficacy and persistence of CAR T cells in patients. Finally, we modified the CAR T cell manufacturing procedure to promote the expansion of more persisting T cells, such as memory T cells.

We confirm the expressions of FOLR1 in OvCa and non-malignant tissue samples. We describe the generation of FOLR1-directed CAR T cells that can efficiently and specifically target OvCa cells in vitro and eradicate a cell line-derived xenograft in vivo. To promote clinical translation, we set up advanced in vitro assays reflecting the suppressive impact of the tumor microenvironment. Likewise, our FOLR1-targeting CAR T cells performed successfully in these advanced in vitro assays.

## 2. Materials and Methods

### 2.1. Patients

#### 2.1.1. Primary Human Blood and Tissues

High-grade serous OvCa patient samples were provided by Prof. Dr. Peter Mallmann (Department of Obstetrics and Gynaecology, Faculty of Medicine, University of Cologne) or purchased from BioIVT and used with informed consent of the patients. Fresh-frozen healthy organ tissue samples were purchased from BioIVT and ProteoGenex.

#### 2.1.2. Ethics Approval

For all studies using human primary tissue from OvCa, written informed consent was obtained following the guidelines of the approved University Hospital Cologne Review Board protocol, respectively. Healthy whole blood samples were taken from donors who had given their written consent beforehand. Peripheral blood mononuclear cells (PBMCs) were isolated from buffy coats of healthy anonymous donors that were purchased from the German Red Cross Dortmund. Leukapheresis products were obtained from the University Hospitals in Cologne and Ulm. All blood samples were handled following the required ethical and safety procedures. All animal experiments were approved under Az: 81-02.04.2022.A196 by the Governmental Review Committee on Animal Care in North Rhine-Westphalia, Germany, and performed according to guidelines and regulations.

### 2.2. Methods

#### 2.2.1. Tissue Processing for Microscopy

The used method for tissue processing was recently described [19]. Briefly, frozen embedded tissue specimens were cryosectioned with a CM3050 cryostat (Leica) with 8 µm per section. Sections were either fixed in pre-chilled acetone  (−20 °C) for 3 min. Subsequently, slides were stored at −80 °C. Sectioned slides were thawed in cold acetone (−20 °C) for 10 min. After air drying, a MACSwell Imaging Frame was mounted on the slide, and MACSima Running Buffer was added (according to the MACSwell Imaging Frames data sheet). Alternatively, cryosectioned slices were directly stored at −80 °C on slides for PFA fixation. Frozen slides were incubated in a 4% PFA solution for 10 min at ambient temperature. Slides washed three times using MACSima Running Buffer (Miltenyi Biotec, Bergisch Gladbach, Germany). After washing, a MACSwell Imaging Frame was mounted on the slide and MACSima Running Buffer was added (according to the MACSwell Imaging Frames data sheet). A 10 min staining with 1:10 diluted 4′,6-diamidino-2-phenylindole (DAPI) was performed before imaging on the MACSima Instrument. Slides were washed three (MACSima Running Buffer), and MACSima Running Buffer was added.

#### 2.2.2. Cyclic Immunofluorescence Staining with the MACSima Imaging Platform

We have reported the use of the MACSima Imaging platform [19]. The MACSima Imaging System is a fully automated instrument including liquid handling and widefield microscopy for cyclic immunofluorescence imaging. In brief, each individual staining cycle is composed of the following (automated) steps: immunofluorescent staining, sample washing, imaging, and signal erasure (photobleaching or removal of REAlease Reagents).

Antibody conjugates used for cyclic immunofluorescence staining with the MACSima Imaging Platform are listed in Supplementary Table S1.

#### 2.2.3. MACSima Data Analysis

Tumor marker FOLR1 expression was quantified on a single-cell level on primary OvCa tissue. Image data sets were segmented using MACS^®^ iQ View Analysis Software (version 1.2) based on nuclei, i.e., DAPI signal and epithelial cell membrane markers, e.g., EPCAM, identifying individual cells. Single cells were analyzed for EPCAM and FOLR1 expressions. Each data point represents single cells from multiple regions of interest.

#### 2.2.4. Generation of CAR Plasmids

Plasmids encoding the CAR constructs were prepared using standard molecular biology and cloning techniques. They all comprised humanized single-chain variable fragments (scFvs) derived from clinical antibodies MORAb-003 and M9436A specific for FOLR1. scFvs were used in both possible orientations (Vh-Vl or Vl-Vh) and connected with a (G_4_S)_3_ linker. Each scFv combination was followed by either an IgG4 or CD8α hinge. The eight resulting MB-CART FOLR1 constructs were designed with CD8α transmembrane domain, 4-1BB costimulatory, and CD3ζ signaling domains. When not specified otherwise, the CAR sequence was linked via a Furin-P2A sequence to induce co-expression of truncated LNGFR.

#### 2.2.5. Generation of CAR T Cells

The generation of CAR T cells was previously reported [20]. In brief, PBMCs were isolated by density gradient centrifugation from whole blood. Thereafter, T cells were purified using the Pan T Cell Isolation Kit, human (Miltenyi Biotec, Bergisch Gladbach, Germany), and T cells were activated in TexMACS™ Medium (Miltenyi Biotec) supplemented with T Cell TransAct™ (Miltenyi Biotec), 12.5 ng/mL of recombinant human interleukin IL-7, and 12.5 ng/mL of recombinant human IL-15 (Miltenyi Biotec). After 24 h of activation, T cells were transduced with vesicular stomatitis virus glycoprotein G (VSV-G) pseudotyped lentiviral particles. Three days after activation, T cells were washed and cultured with fresh TexMACS™ Medium supplemented with 12.5 ng/mL of recombinant human interleukin IL-7 and 12.5 ng/mL of recombinant human IL-15. On day 12 after transduction, CAR T cells were used in in vitro assays, and the number of viable CAR T cells was determined by staining T cells with 7-AAD (Miltenyi Biotec) and biotinylated human FOLR1 protein (Miltenyi Biotec). To generate CAR T cells from patient-derived ascites, the ascites suspension was centrifuged for 5 min at 300 g to separate the cellular fraction. T cells were transduced within the total cellular fraction of ascites. Transduction, as well as culture, was performed as described above.

#### 2.2.6. Cell Lines and Culture Conditions

Human embryonic kidney 293T cells (HEK293T, ACC 635) were obtained from the DSMZ—German Collection of Microorganisms and Cell Cultures. OV-90 (CRL-11732), Caov-3 (HTB-75), NIH:OVCAR-3 (HTB-161), and SKOV-3 (HTB-77) cells were obtained from ATCC and cultured as recommended. Jurkat E6 cells were cultured at 37 °C, 5% CO_2_ in RPMI (Biowest, Riverside, MO, USA) with 10% FCS (Eximus, Melville, NY, USA) and 5mM L-glutamine (Biowest). Cell lines were routinely analyzed for mycoplasma contamination.

#### 2.2.7. Cell Line Generation

FOLR1 knockout cells were generated by CRISPR/Cas9-mediated knockout and followed by flow cytometric single-cell sorting and additional single-cell cloning. Ribonucleoprotein particle complexes were assembled according to the manufacturer’s protocol (IDT, Coralville, Iowa, USA). Briefly, CRISPR-Cas9 gRNAs targeting the human FOLR1 locus (CCTACCTATATAGATTCAACTGG) were incubated with CRISPR-Cas9 crRNA for 5 min at 95 °C at a 1:1 ratio, and the mixture was allowed to form gRNA complexes by cooling down at ambient temperatures for 20 min. Next, gRNA complexes were incubated with Cas9 nuclease at a 1:1 ratio for 20 min at ambient temperature to assemble into ribonucleoprotein particle complexes. Thereafter, 1 × 10^6^ OV-90 cells were mixed in 40 µL electroporation buffer (Miltenyi Biotec), including 10 µL ribonucleoprotein particle complexes (equals 2.7 µg ribonucleoprotein particle complexes). The mixture was electroporated with the CliniMACS Electroporator (Miltenyi Biotec). Subsequently, FOLR1 knockout cells were sorted with the MACSQuant Tyto Cell Sorter (Miltenyi Biotec). Knockout efficiency was monitored by flow cytometry, qPCR, microscopy, and gDNA sequencing to assess FOLR1 expression. Single-cell cloning was performed by serial dilution.

FOLR variants expressing Jurkat cells were modified by lentiviral transduction. Lentiviral particles expressing human FOLR1 (gene ID: 2348), human FOLR2 (gene ID: 2350), human FOLR3 (gene ID: 2352), human FOLR4 (gene ID: 390243), murine FOLR1 (gene ID: 14275), murine FOLR2 (gene ID: 14276), or murine FOLR4 (gene ID: 64931), respectively.

#### 2.2.8. Spheroid Generation

Spheroid generation was performed as recently described [21]. Briefly, Matrigel^®^ was thawed overnight at 4 °C and subsequently added to 4 °C cooled OV-90 medium at a ratio of 1:50. The cells were added to the medium mixture to achieve a cell concentration of 20,000 cells/mL. From the cell suspension, 100 μL were added per well of a 96-well U-shaped ultra-low attachment plate (2000 cells/well). Cells were then cultured at 37 °C, 5% CO_2_ for 2–6 d. For incubation beyond 2 d, an additional 200 μL of OV-90 growth medium was added to the cells.

#### 2.2.9. Manufacturing of CAR T Cells with the CliniMACS Prodigy

Automated MB-CART-FOLR1 production with the CliniMACS Prodigy™ system (Miltenyi Biotec) using the TCT process was performed as described over 12 d [22]. Where not specified otherwise, reagents and materials were obtained from Miltenyi Biotec. To start the process, CD4/CD8 positive T cells were automatically enriched from a fresh leukapheresis in CliniMACS PBS/EDTA process buffer supplemented with 0.5% Human Serum Albumin (Octapharma, Lachen, Switzerland). After magnetic CD4/CD8 enrichment, 1 × 10^8^ T cells were transferred to the culture chamber on the same day and stimulated for 24 h with TransAct™. T cells were transduced with anti-FOLR1 CAR lentiviral vectors (LV). After 5 d of cultivation in TexMACS GMP medium including 12.5 ng/mL of human recombinant IL-7 and IL-15 as well as 3% heat-inactivated human AB serum (Capricorn Scientific, Ebsdorfergrund, Germany), the medium was changed to serum-free IL-7- and IL-15-supplemented TexMACS GMP medium. In-process controls were performed to monitor CAR T cell expansion, including MACSQuant^®^ Analyzer 16-based flow cytometric analysis, using express mode panels A and B to determine cell count and CAR frequency.

#### 2.2.10. Cytotoxicity Assays

Target cells were seeded in triplicates in 96-well culture plates at densities of 2 × 10^4^ GFP-expressing OvCa cells or 1.5 × 10^4^ Jurkat cells per well in their respective culture medium. CAR T cells were added 18 h later at an effector-to-target cell ratio (E/T) of 2:1. Final vessel volume was adjusted to 200 μL. The total amount of T cells was adjusted to the number of total T cells in the CAR group with the lowest transduction efficiency. Cytotoxicity was measured as a decrease in green surface area with an IncuCyte^®^ S3 Live-Cell Analysis System (Sartorius, Goettingen, Germany) and the respective software IncuCyte S3 (v2017A, v2018C, and v2019A). The change in green surface area was calculated relative to the starting value. After 48 h, at the end of co-culture, CAR T cells were analyzed with a flow cytometer for expression of activation markers (CD25, CD69, 41BB), exhaustion markers (TIM3, LAG3, PD1), and phenotype markers (CD62L, CD45RO, CD45RA). To detect intracellular Granzyme B, cells were incubated with Brefeldin A 1 µg/mL (Sigma-Aldrich, Taufkirchen, Germany) 24 h prior to staining at 37 °C. Intracellular staining was performed using the Inside Stain Kit (Miltenyi Biotec). In addition, after 24 h, 50 μL of medium was collected and used for analysis of secreted cytokines with the MACSPlex Cytokine 12 Kit, human (Miltenyi Biotec).

#### 2.2.11. Flow Cytometry

MACSQuant^®^ Analyzer 10 or 16 were used to measure all samples, and analysis was performed using the MACSQuantify™ Software v2.13.0/v2.11.0 or Flowlogic. Antibody conjugates that were used for staining of surface marker expression are listed in Supplementary Table S1. Stainings performed with antibody conjugates from Miltenyi Biotec were performed as recommended by the manufacturer. Antibodies from Biolegend, San Diego, CA, USA were applied at a concentration of 5 µg/mL and incubated for 10 min at 4 °C. Subsequent to the incubation, a washing step was performed adding PEB (phosphate-buffered saline (PBS) + 0.05% EDTA + 10% bovine serum albumin (BSA)). Cells were spun down, resuspended in PEB, and measured on a flow cytometer. Dead cells were excluded using PI or 7-AAD.

#### 2.2.12. qPCR and VCN Determination

To perform qPCR and VCN determination, 1.0 × 10^6^ of CAR positive cells were spun down at 300 g for 5 min and stored at −20 °C until further processing. On the day of VCN determination, genomic DNA was isolated using the DNeasy Blood & Tissue Kit (Qiagen) following the manufacturer’s instructions. Afterward, qPCR was performed using the MACSCOPYcheck Kit (Miltenyi Biotec) again following manufacturer’s instructions. VCN calculation formula was also provided by the MACSCOPYcheck Kit.

#### 2.2.13. gDNA Sequencing

Genomic DNA was isolated using DNeasy Blood & Tissue Kit (Qiagen, Hilden, Germany). FOLR1 sequence was subsequently amplified via PCR using Q5 Hot Start High-Fidelity 2X Mastermix (New England Biolabs, Ipswich, MA, USA) and FOLR1 flanking primer pair (forward primer sequence: 5′-AGGGAGGGGTGGTGTCTAAT-3′; reverse primer sequence: 5′-CCTTTGGGCCTGCTTCCTTA-3′ (Metabion, Planegg, Germany). PCR product was afterward cleaned via the NucleoSpin Gel and PCR Clean-Up Kit (Qiagen). The purified PCR product was sent to Eurofins genomics for sequencing.

#### 2.2.14. In Vivo Experiments

All animal experiments were approved under Az: 81-02.04.2022.A196 by the Governmental Review Committee on Animal Care in North Rhine-Westphalia, Germany, and performed according to guidelines and regulations. Mice were housed in IVC stations at ambient temperature with dark/light cycle of 12/12 h and humidity between 45% and 65%.

Cell line-derived xenografts (CDXs) were generated by injection of 1 × 10^7^ OV-90 or OV-90 *FOLR1* knockout cells s.c. into the right flank of NOD SCID gamma (NSG; NOD.CgPrkdcscidIl2rgtm1Wjl/SzJ) mice (Jackson Laboratory, Bar Harbor, ME, USA, provided by Charles River). Tumors were established for 21 days. CAR T cells (1 × 10^7^ CAR T cells in 100 µL PBS) were applied into the tail vein. Mock T cell numbers were adjusted to the highest concentration of total T cells in all groups. Anti-tumor response was measured longitudinally. D-Luciferin (GoldBio) was intraperitoneally injected (100 μL of a 30 mg/mL D-Luciferin solution). Subsequently, an in vivo imaging system (PerkinElmer, Waltham, MA, USA) and the supplied software Living Image (v4.7.3) were used to visualize tumor size. Well-being of mice was secured by following the relevant animal use guidelines. Flow cytometric analyses of bone marrow, spleen, and tumor were performed after dissociation using the gentleMACS™ Octo Dissociator with heaters according to the manufacturer’s protocol (Miltenyi Biotec) and red blood cell lysis using Red Blood Cell Lysis Solution (Miltenyi Biotec).

#### 2.2.15. Light Sheet Microscopy

Samples were prepared based on an immunostaining and tissue-clearing protocol according to manufacturer’s protocol (Miltenyi Biotec). After fixation, spheroids were kept in 200 µL of permeabilization solution (MACS^®^ Clearing Kit) for 6 h. Permeabilization solution was replaced with antibody staining solution (MACS^®^ Clearing Kit), containing either Vio^®^ R667 or Vio^®^ R570 conjugated anti-CD3 antibody at a concentration of 5 µg/mL. Antibodies were incubated for 12 h at 37 °C. Samples were washed three times with antibody staining solution (MACS^®^ Clearing Kit) for 30 min each. Next, samples were embedded in 1.5% agarose blocks. For dehydration, ethanol with 2% Tween^®^ 20 was used. First, agarose blocks were incubated in 50% alcohol for 2 h with constant rotation, followed by additional 2 h in 70% ethanol. Samples were placed in 100% alcohol and further incubated overnight. To render the samples transparent, they were transferred into the Clearing Solution (MACS^®^ Clearing Kit). After 3 h, this solution was replaced with fresh Clearing Solution and incubated for an additional 3 h. After clearing, the samples were ready for 3D imaging using the UltraMicroscope Blaze™ (Miltenyi Biotec, Bergisch Gladbach, Germany).

Once the respective markers were stained and the sample cleared, the agarose blocks were mounted on a slide in the chamber of the UltraMicroscope Blaze™. Depending on the dye or fluorochrome, the respective channels with specific excitation lasers and emission filters were used. Based on the brightness of the dye, the laser power was regulated between 10% and 1%, with a constant exposure time of 300 ms. Z-stacks with a spacing of 10 µm were taken with either the 4× or 12× objective and an internal zoom of 1×. For operating the UltraMicroscope Blaze™, the software ImSpector (version 7.6.0) was used.

The software tools Fiji (version 1.53) [23] and Imaris were used for the depiction and cropping of the 3D images. Furthermore, Fiji was used to adjust brightness and contrast as well as to measure distances and sizes within the samples. An in-house programmed macro for Fiji allowed us to calculate the signal mean intensity in relation to the background intensity. In short, first a threshold was defined, above which the signal intensity was valued. Afterward, artifacts that could be clearly differentiated from the actual sample were manually excluded from the calculation. After defining the background, the macro calculated an average brightness value over the entire selected area. Five planes per sample containing up to six spheroids were evaluated, with homogeneous distribution through the spheroid. This way, it was possible to convert the fluorescence images into quantifiable values.

#### 2.2.16. Statistical Analysis

Unless otherwise specified, all graphs show the mean with error bars representing the standard error of the mean. Statistical comparisons were conducted with GraphPad Prism 8. For all mouse experiments, the number of independent mice used is stated in the figure legend.

## 3. Results

### 3.1. FOLR1 Is Differentially Expressed in Ovarian Carcinoma and Healthy Tissues

**Figure 1:**
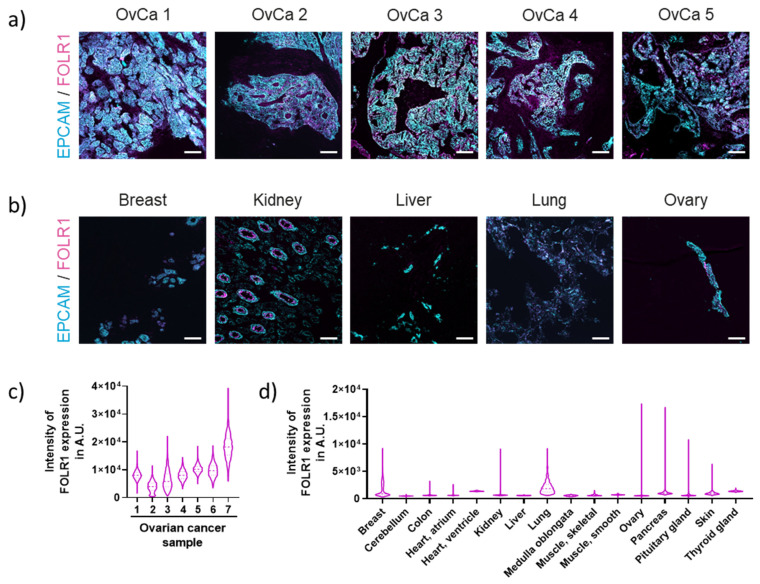
Ultrahigh-content imaging enables the identification of FOLR1 as potential target for high-grade serous epithelial OvCa. (**a**) Co-expression of EPCAM (cyan) and FOLR1 (magenta) in HGSOC samples (each number indicates an individual patient sample). Individual stainings are shown in Supplementary Figure S1a. Scale bar represents 100 µm. (**b**) Co-expression of EPCAM (cyan) and FOLR1 (magenta) in a selection of healthy tissues. Individual stainings and additional healthy samples are presented in Supplementary Figure S1b,c. Scale bar represents 100 µm. (**c**) Quantification of FOLR1 expression on a single-cell level on epithelial cells from primary OvCa tissue. (**d**) Quantification of FOLR1 expression on a single-cell level on epithelial cells from healthy tissue.

We have previously reported the successful identification of novel tumor targets for different solid cancers, employing the ultrahigh-content imaging platform MACSima^TM^ [18,20]. Here, we used the same ultrahigh-content imaging platform to identify target molecules for CAR T cell therapy in OvCa. Tissue samples from high-grade serous ovarian carcinoma patients were analyzed with a cell surface marker library to identify the expressions of tumor markers. Among tested antigens, FOLR1 expression was most consistently detected in tumor cells. FOLR1 expression was detected in the epithelial tumor cells of all patient samples, indicated by co-expression with EPCAM (Figure 1a; Supplementary Figure S1a,b), which is a well-known marker for human epithelial tissues and carcinomas [24].

To assess the safety of FOLR1 as a CAR T cell target, healthy tissues were also evaluated for the off-tumor expression of FOLR1 (Figure 1b; Supplementary Figure S1c–f). In addition to co-expression with EPCAM, FOLR1 expression levels were elevated, as indicated by the quantified intensities among the OvCa tissues analyzed (Figure 1c). In contrast, FOLR1 expression was lower in healthy tissues compared to OvCa tissues (Figure 1 c,d). We validated that the expression of FOLR1 is low or absent in all tissues analyzed [7]. Low FOLR1 expression was detected in several known tissues, including breast, kidney, and lung. In the kidney, we could verify restricted FOLR1 localization to the apical surface of healthy cells, rendering the receptor potentially inaccessible to blood-circulating drugs (Figure 1b) [25]. These data highlight the promising safety profile of FOLR1 as a target antigen for CAR T cells. Thus, we decided to develop novel CAR T cells against FOLR1.

### 3.2. Characterization of Anti-FOLR1 CAR Designs Reveals Efficient and Specific CAR Candidates In Vitro

**Figure 2:**
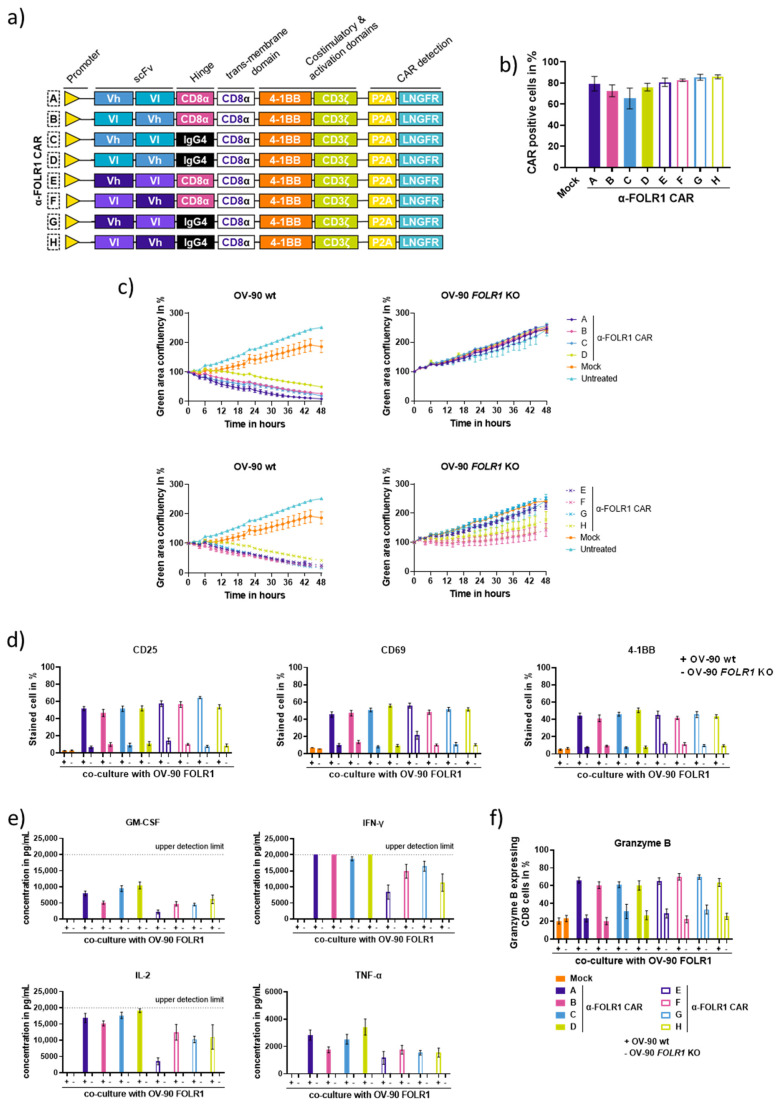
In vitro evaluation of novel CAR T cell candidates. (**a**) Schematic diagram of the anti-FOLR1 CAR library designed with varying hinges (IgG4 or CD8α), scFv sequences, and scFv orientations. FOLR1 binding scFvs are derived from either MORAb-003 (anti-FOLR1 CAR candidates A–D) or M9346A4 (anti-FOLR1 CAR candidates E–H). (**b**) Anti-FOLR1 CAR expression was detected via anti-LNGFR staining and flow cytometry. Each bar represents the mean ± SEM of three donors (*n* = 3). (**c**) Representative killing kinetics of the different anti-FOLR1 CAR T cell candidates with OV-90 wt or *FOLR1* KO cells in co-culture at an effector-to-target ratio of 2:1 (additional T cell donors are shown in Supplementary Figure S3). Each data point represents mean ± SEM (*n* = 3). (**d**) Activation markers CD25, CD69, and 4-1BB expressed by CAR T cells after 48 h of co-culture assay. (**e**) Secreted cytokines GM-CSF, IFN-γ, IL-2, and TNF-α after 24 h of co-culture with OV-90 wt or *FOLR1* KO cells. (**f**) Expressions of Granzyme B by anti-FOLR1 CAR T cells were analyzed after 24 h of co-culture with OV-90 wt or *FOLR1* KO cells. Intracellular Granzyme B was detected via flow cytometry. Each bar represents the mean value ± SEM of three replicates from three donors (*n* = 9).

Since the CAR design has an impact on the functionality and specificity of the CAR T cell [26,27], we investigated different CAR constructs to identify the most promising anti-FOLR1 CAR candidate. Second-generation CAR constructs were designed with a CD8α transmembrane domain, human 4-1BB costimulatory domain, and human CD3ζ domain. The FOLR1 binding sequences were derived from monoclonal antibodies, MORAb-003 and M9346A4, respectively, and converted to the single chain fragment variable (scFv) domain of the CAR. We assessed different orientations of the heavy (Vh) and light (Vl) chains within the scFv, i.e., Vh-Vl and Vl-Vh, as well as different hinge domains linking the scFv to the transmembrane domain of the CAR, CD8α, and IgG4 hinges, respectively. Additionally, each CAR construct co-expressed a truncated low-affinity nerve growth factor receptor (LNGFR) as a reporter gene and surrogate marker for CAR expression (Figure 2a) [28]. The eight different CAR constructs were transduced in T cells from healthy donors, and the expression of the corresponding CAR was assessed by the flow cytometry staining of LNGFR (Figure 2b).

To evaluate the functionality and specificity of the different CAR candidates, co-culture experiments with a human OvCa cell line (OV-90) expressing GFP and luciferase were performed. The OV-90 cells were either wild-type FOLR1-proficient (wt) or FOLR1-deficient via CRISPR/CAS9-mediated knockout (*FOLR1* KO). Homozygous FOLR1 knockout was confirmed by sequencing of genomic DNA (Supplementary Figure S2a), qPCR (Supplementary Figure S2b), immunofluorescence (Supplementary Figure S2c), and flow cytometry (Supplementary Figure S2d). Co-culture experiments of the eight CAR candidates with OV-90 wt cells revealed efficient killing kinetics (Figure 2c left panel; Supplementary Figure S3a). However, candidates D and H (Vl-Vh-oriented scFv, IgG4 hinge) showed reduced in vitro cytolytic capacity (Figure 2c left panel). It is worth noting that most candidates did not control the growth of OV-90 cells deficient for FOLR1, except for candidates F and H, indicating antigen-independent cytolytic activity (Figure 2c right panel; Supplementary Figure S3b).

Anti-FOLR1 CAR T cells were analyzed for the expression of activation marker expression and cytokine secretion upon co-culture with OV-90 cells. Activation markers CD25, CD69, and 4-1BB were induced at similar levels in an antigen-dependent manner for all eight CARs after 48 h of co-culture (Figure 2d). Analysis of exhaustion marker expression, i.e., TIM3, LAG3, and PD1, showed similar expression levels for most candidates (Supplementary Figure S3c). However, candidates E and F presented with higher frequencies of PD1 and LAG3 double-positive cells. T cells expressing anti-FOLR1 CAR candidate F express more frequently all three exhaustion markers TIM3, LAG3, and PD1 after co-culture with OV-90 cells. The co-culture medium was analyzed for secreted cytokines after 24 h (Figure 2e). All CAR T cell candidates secreted cytokines GM-CSF, IFN-ɣ, IL-2, and TNF-α antigen dependently. The anti-FOLR1 CAR T cell candidates A-D tended to secrete more cytokines than candidates E–H. Additionally, FOLR1-directed CAR T cell candidates specifically expressed comparable levels of Granzyme B (Figure 2f). CAR T cell candidates were also analyzed for their phenotype after co-culture with OV-90 and OV-90 *FOLR1* KO cells (Supplementary Figure S3d,e). After co-culture with FOLR1-proficient target cells, CD8^+^ CAR T cells had an effector memory T cell (T_em_) phenotype, whereas the co-culture of the CAR T cells with FOLR1-deficient cells remained in a stem cell-like memory T cell (T_SCM_) phenotype. This suggests that the antigen engagement of the anti-FOLR1 CAR led to T cell differentiation.

FOLR1 is a member of the folate receptor family, which has a high degree of similarity [29]. To assess the potential cross-reactivity of FOLR1-directed CAR T cells towards other folate receptors, we generated Jurkat cell lines expressing human or murine FOLR variants (Supplementary Figure S4a), respectively. To assess the specificity of the CAR T cell candidates for hFOLR1, each of the different FOLR-expressing Jurkat cell lines was co-cultivated with the different anti-FOLR1 CAR candidates. Subsequently, CAR T cells were analyzed for the expression of activation markers, CD25, CD69, and 4-1BB as indicators for CAR T cell reactivity (Supplementary Figure S4b). None of the tested CAR constructs showed reactivity to any FOLR variant other than human FOLR1, suggesting that the developed CAR T cell candidates are highly specific for human FOLR1.

Due to strong in vitro killing efficacy among several donors accompanied by antigen-dependent activation marker expression and cytokine secretion, we selected candidates with scFv in Vh-Vl orientation for subsequent in vivo validation, i.e., anti-FOLR1 CAR candidates A, C, E, G, respectively.

### 3.3. Rapid In Vivo Eradication of Advanced OV-90 Xenografts by Anti-FOLR1 CAR T Cells Depends on the CAR Design

**Figure 3:**
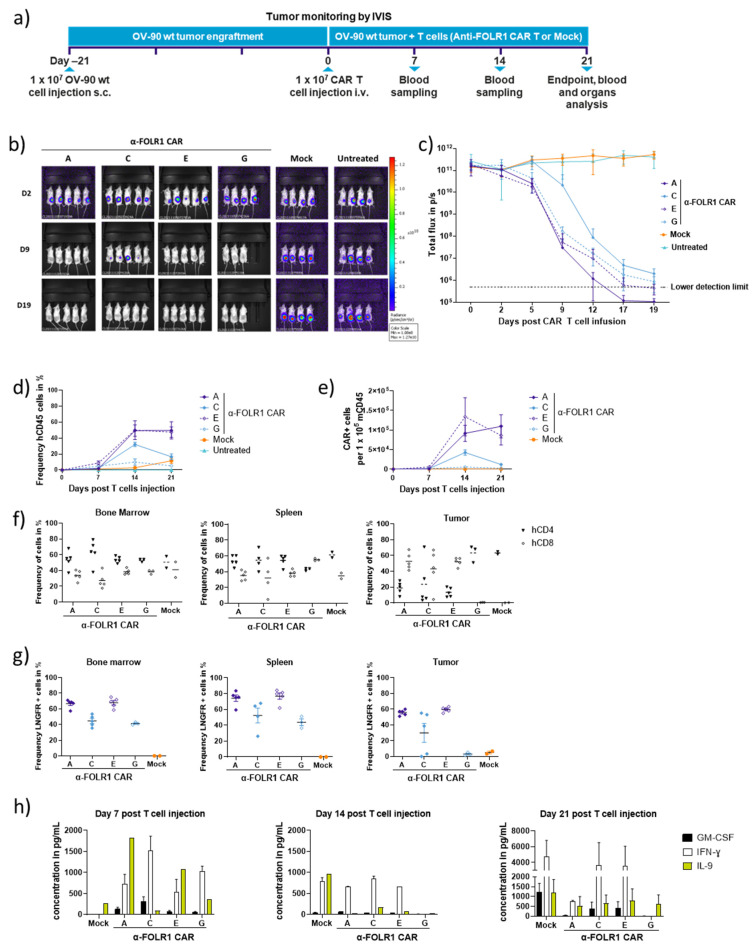
Anti-FOLR1 CAR T candidates efficiently eradicate OV-90 wt tumors in vivo. (**a**) Scheme of this study evaluating intravenously injected CAR T cell efficacy against subcutaneous OV-90 wt xenograft tumors. (**b**) Representative bioluminescence images of individual OV-90 xenograft tumor-bearing NSG mice post CAR T cell injection. (**c**) Quantification of bioluminescence as total flux in photons per second (p/s) of the different experimental groups after CAR T cell injection over 21 d. Data points represent mean ± SEM. Human leucocyte expansion in peripheral blood of injected mice was analyzed by flow cytometry over time. (**d**) Human CD45 expression and (**e**) CAR-positive cells relative to mouse CD45 cells in blood samples collected at day 7, 14, and 21 post T cell injection were measured. Data are shown as mean ± SEM. On day 21 post T cell injection, mice were sacrificed, and the respective organs were collected and dissociated for ex vivo analysis by flow cytometry. (**f**) Human CD4 and CD8 frequencies as well as (**g**) CAR expression (detected as LNGFR positive) among human CD45 in mouse bone marrow, spleen, lung, and tumor were analyzed, respectively. Each data point represents an individual mouse and horizontal lines represent the mean of the respective group. (**h**) Secretion of human cytokines was analyzed in peripheral blood samples over time. Plasma was isolated from blood samples collected on days 7, 14, and 21 post T cell injection, and cytokine levels were subsequently determined with the human MACSplex Cytokine 12 Kit. Data are shown as mean ± SEM.

We investigated the in vivo anti-tumor efficacy of four selected anti-FOLR1 CAR T cell candidates A, C, D, and F, respectively. NSG mice were subcutaneously injected with OV-90 cells expressing GFP and luciferase, and after 21 d of tumor establishment, a single dose of CAR T cells was injected intravenously (1 × 10^7^ CAR T cells) to assess anti-tumor efficacy over a period of an additional 21 d, including weekly blood withdrawals. At the end of this study, the distribution of CAR T cells in selected organs and xenograft tumors was analyzed (Figure 3a). The CAR T cell products were generated with the automated cell processing system CliniMACS Prodigy^®^. All CAR T cell products were composed of more than 96% T cells with high viabilities of 97%. Transduction efficiency was determined at 55%.

All CAR candidates efficiently reduced tumor burden within 21 d post injection of CAR T cells (Figure 3b,c). However, the tested anti-FOLR1 CAR candidates differed in their in vivo performance. FOLR1-specific CAR candidates designed with IgG4 hinge (candidates C and G) are associated with slower tumor-killing kinetics, reduced proliferation capacities, and persistency compared to the CAR candidates designed with CD8α hinge (candidates A and E) (Figure 3b–e).

At the end of this study, we detected human and murine leukocytes (Supplementary Figure S5a), human CD4 and CD8 T cells (Figure 3f), as well as CAR T cells (Figure 3g), in the spleen, bone marrow, and xenograft tumors. These findings suggest that anti-FOLR1 CAR T cells can infiltrate into the tumor and, subsequently, home to the spleen and bone marrow. Additionally, we measured human cytokines in blood samples weekly (Figure 3h, Supplementary Figure S5b).

It is worth noting that anti-FOLR1 CAR candidate C induced a temporary weight loss (Supplementary Figure S5c) correlating with delayed tumor elimination (Figure 3b,c) and higher IFN-γ and GM-CSF secretion (Figure 3h). Seven days post T cell injection, we observed the recovery of weight, cytokine levels, and tumor killing (Figure 3c,h; Supplementary Figure S5c). This temporary weight loss was confirmed in tumor-free mice after the injection of anti-FOLR1 CAR candidate C (Supplementary Figure S5d), indicating an off-target in vivo reactivity. Anti-FOLR1 candidate G also showed no detectable proliferation (Figure 3d,e) and no detectable tumor infiltration by CAR^+^ and CD8 cells (Figure 3f,g).

On the other hand, candidates A and E showed superior anti-tumor efficacy (Figure 3b,c) accompanied by an expansion of human leukocytes (CD45 cells) and CAR-T cells (Figure 3d,e). Interestingly, anti-FOLR1 candidates A and E showed an increased presence of human CD45, CAR^+^, and CD8 T cells in the tumor tissues (Figure 3f,g; Supplementary Figure S5a). Analysis of cytokine secretion during the 21 d post CAR T cell infusion revealed a moderated and stable secretion of IFN-γ, which returned to basal levels after 21 d for candidate A (Figure 3h). Interestingly, we observed an increase in IL-9 at day 7 post CAR T cell injection for anti-FOLR1 CAR candidates A and E, correlating with the faster tumor-killing kinetics. IL-9 is a pleiotropic cytokine expressed by many cells, including activated T cells, and it may exert growth factor and anti-apoptotic functions [30]. Although the different functions of IL-9 are still under debate, we observed a temporary increase in IL-9 on day 7 post CAR T cell injection, correlating with better anti-tumor responses of candidates A and E.

Thereby, we concluded that anti-FOLR1 CAR candidates A and E performed best in vivo, indicated by rapid tumor eradication, pronounced proliferation, CAR expression, and short-term persistence. Moreover, anti-FOLR1 CAR candidates A and E showed pronounced homing to bone marrow and spleen, and superior tumor infiltration compared to other CAR candidates.

### 3.4. Optimized Anti-FOLR1 CAR T Cells Are Functional In Vitro

**Figure 4:**
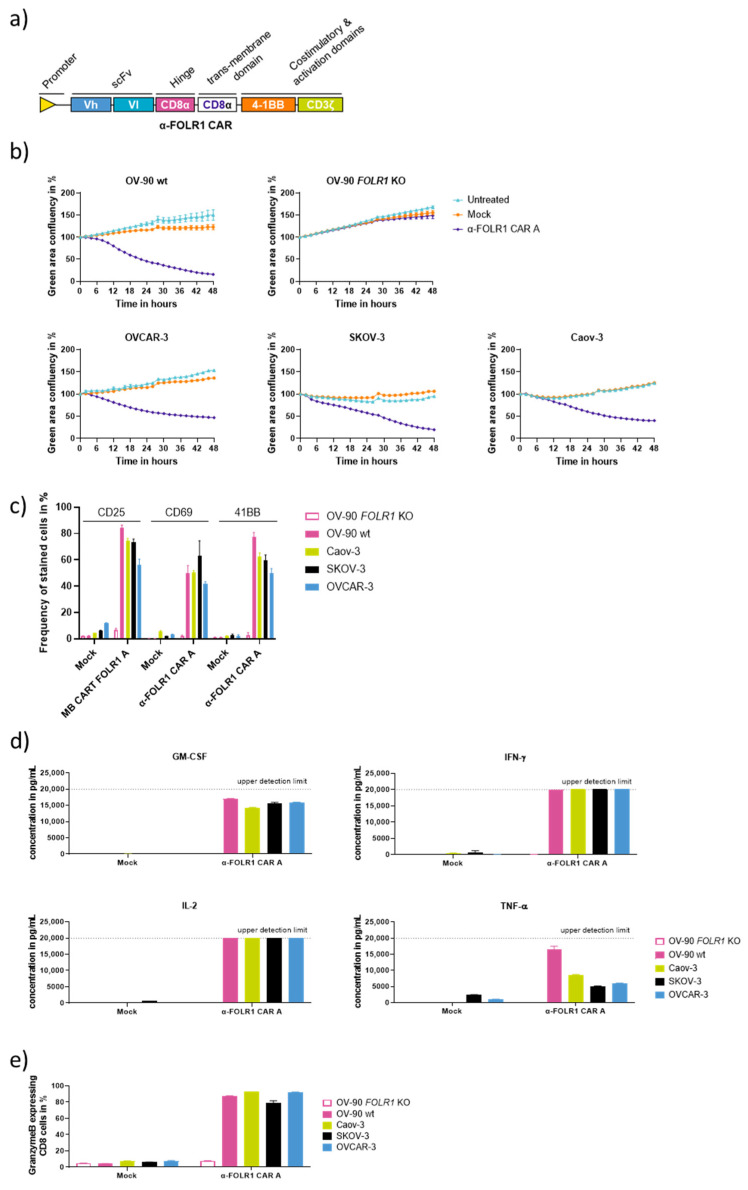
In vitro evaluation of optimized anti-FOLR1 CAR T cells. (**a**) Schematic diagram of the second generation anti-FOLR1 CAR T cell designed with CD8α hinge and CD8α transmembrane domain and scFv in Vh-Vl orientation without truncated LNGFR reporter gene. Anti-FOLR1 CAR candidate A or untransduced T cells (Mock) produced with the CliniMACS Prodigy^®^ TCT process were co-cultured for 48 h with OvCa cell lines at an effector cell-to-target cell ratio of 2:1. (**b**) Co-culture experiments of GFP-expressing target cell lines OV-90 *FOLR1* KO, OV-90 wt, Caov-3, SKOV-3, and OVCAR-3 with anti-FOLR1 CAR T cell candidate A. Data points of each group are normalized to baseline (defined as 100% confluency at the first timepoint (T = 0 h)), and each data point represents mean ± SEM (*n* = 3). Anti-FOLR1 CAR T cells express various levels of activation markers. (**c**) After 48 h of co-culture with the different target cells, CAR T cells were assessed by flow cytometry for expressions of activation markers CD25, CD69, and CD137. Each bar represents the mean ± SEM (*n* = 3). (**d**) Anti-FOLR1 CAR T cells antigen-dependently secrete cytokines in co-culture with OV-90 wt, Caov-3, SKOV-3, and OVCAR-3 cells, whereas OV-90 *FOLR1* KO cells did not induce cytokine secretion by CAR T cells. Supernatants were collected after 24 h of co-culture, and cytokine concentration was subsequently determined with the human MACSplex Cytokine 12 Kit. Each bar represents the mean ± SEM (*n* = 3). (**e**) Anti-FOLR1 CAR T cells express Granzyme B antigen dependently in co-culture with OV-90 wt, Caov-3, SKOV-3, and OVCAR-3 cells, whereas OV-90 *FOLR1* KO cells did not induce Granzyme B expression in CAR T cells. T cells were collected after 24 h of co-culture and overnight block of protein secretion by incubation with Brefeldin A (1 μg/mL). Cells were subsequently fixed, stained for CD8 surface marker, and permeabilized, and finally, intracellular Granzyme B was detected via flow cytometry. Each bar represents the mean ± SEM (*n* = 3).

Prompted by the in vitro as well as the in vivo performance of anti-FOLR1 CAR candidate A, we decided to focus on this construct. To further adapt the CAR constructs to clinical requirements, anti-FOLR1 CAR T cells were designed with an alternative lentiviral vector lacking the LNGFR reporter (Figure 4a). For in vitro characterization, this optimized vector was used to manufacture CAR T cells with the CliniMACS Prodigy^®^. CAR T cells were co-cultured with various OvCa cell lines with varying FOLR1 expression indicated by differences in median fluorescence intensity, i.e., Caov-3, OV-90 wt, OV-90 *FOLR1* KO, OVCAR-3, and SKOV-3, respectively (Supplementary Figure S6). Anti-FOLR1 CAR candidate A efficiently lysed OvCa cells OV-90 wt, OVCAR3, SKOV-3, and Caov-3, whereas OV-90 *FOLR1* KO grew out independently of the presence of CAR T cells in the co-culture (Figure 4b). This suggests that our anti-FOLR1 CAR can target OvCa cells with low to high expressions of FOLR1.

After 48 h of co-culture, CAR T cells were analyzed by flow cytometry to assess activation marker expression (Figure 4c). Activation marker expression on the anti-FOLR1 CAR T cells was induced and correlated with FOLR1 expression on the OvCa cell lines. The co-culture medium was analyzed after 24 h for the presence of human cytokines using the MACSplex^TM^ assay. Anti-FOLR1 CAR candidate A secreted cytokines GM-CSF, IFN-γ, IL-2, and TNF-α antigen-dependently (Figure 4d). The majority of FOLR1-directed CAR T cells expressed Granzyme B in an antigen-dependent way (Figure 4e). Taken together, optimized anti-FOLR1 CAR candidate A performed similarly against various OvCa cell lines in vitro, with efficient and antigen-dependent cytolytic functionality. Therefore, anti-FOLR1 CAR candidate A was further characterized.

### 3.5. Anti-FOLR1 CAR T Cells Infiltrate Ovarian Cancer Spheroids and Lyse Patient Tumor Cells In Vitro

**Figure 5:**
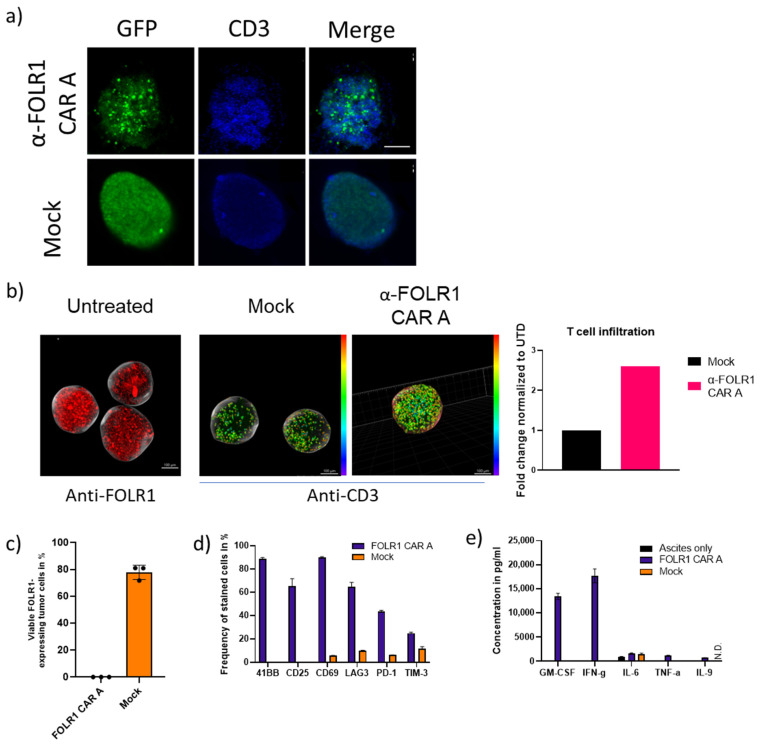
Evaluation of CAR T cells in advanced in vitro assays. (**a**) FOLR1-directed CAR and untransduced T cells were co-cultured with GFP-expressing OV-90 spheroids for 16 h. Spheroids were analyzed for GFP expression and CD3 cell presence. The scale bar represents 100 µm for each condition. (**b**) FOLR1-directed CAR and untransduced T cells were co-cultured with GFP-expressing OV-90 spheroids for 4 h. Spheroids were analyzed by light sheet microscopy for CAR T cell infiltration. Light sheet microscopy of OV-90 (left graph) shows FOLR1 expression in OV-90 spheroid. Middle and right graph color coding for anti-CD3 stains indicates distance of CD3 cells to surface. Number of infiltrated CAR T cells and penetration depth were quantified. Fold change is based on N/µm^3^ measurements. Patient-derived OvCa samples were dissociated, and FOLR1 expression was analyzed by flow cytometry. (**c**) FOLR1-directed CAR T cells were co-cultured with dissociated primary OvCa tumor for 16 h. Thereafter, the frequency of FOLR1-expressing tumor cells was analyzed by flow cytometry. (**d**) After co-culture with OvCa tumor cells, FOLR1 CAR T cells were analyzed for expression of the indicated surface markers by flow cytometry. (**e**) Concentration of secreted cytokines was determined in the co-culture supernatant.

Next, we performed advanced in vitro characterization of our FOLR1-specific CAR T cells to address the influence of immunosuppressive mechanisms present in solid tumors. First, we assessed the capability of our CAR T cells to infiltrate three-dimensional structures, OvCa spheroids. OV-90 spheroids were generated and co-cultured with CAR T cells or untransduced T cells (Mock). FOLR1-specific CAR T cells rapidly disintegrated OV-90 spheroids (Figure 5a). Subsequently, we shortened the co-cultivation time of OV-90 spheroids and CAR T cells and performed light sheet microscopy to quantify the number of CAR T cells infiltrating into the spheroids and the penetration depth of CAR T cells, as indicated by the color coding (Figure 5b). The penetration depth of FOLR1-directed CAR T cells was significantly increased, indicating that anti-FOLR1 CAR T cells can infiltrate into a three-dimensional structure and lyse the respective tumor cells.

Second, we assessed the functionality of our FOLR1-specific CAR T cells against patient-derived tumor cells in an autologous setup, i.e., tumor cells, CAR T cells, as well as ascites derived from the same patient. To simulate the tumor microenvironment in patients, we also replaced the co-culture experiment with the cell culture medium with malignant ascites fluid of the same respective patient where tumor cells were derived from. Ascites has been reported to be immune suppressive [31]. After tumor dissociation, we confirmed approximately 75% FOLR1 expression in the tumor cells (Figure 5c). At the end of co-culture with anti-FOLR1 CAR T cells, patient-derived tumor cells were completely lysed (Figure 5c). CAR T cells expressed activation (4-1BB, CD25, CD69) and exhaustion markers (LAG3, PD1, TIM3) (Figure 5d) complemented by cytokine secretion (Figure 5e). GM-CSF and IFNγ were mainly secreted, whereas only low levels of IL-6, IL-9, and TNFα were detected. It is worth noting that IL-6 was the only cytokine we detected in untreated ascites.

Taken together, anti-FOLR1 CAR T cells can infiltrate into OvCa spheroids and are functional against patient-derived tumor cells in the presence of malignant ascites.

### 3.6. Optimized Anti-FOLR1 CAR T Cells Specifically Reduce Tumor Burden Antigen-Dependently in an Ovarian Cancer Model

**Figure 6:**
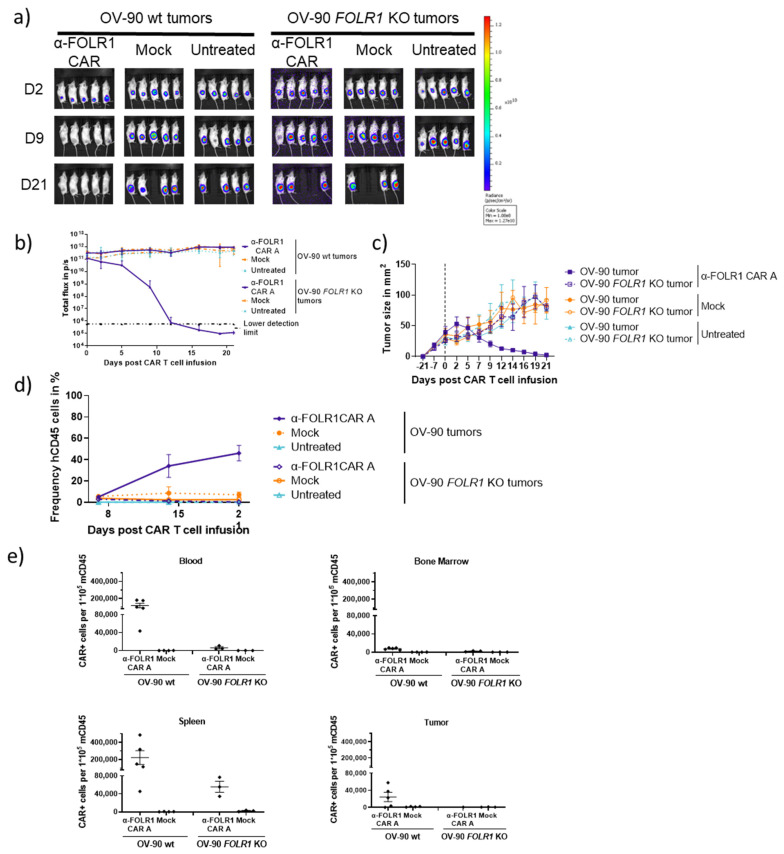
Optimized anti-FOLR1 CAR T cells efficiently and specifically eradicate tumors in vivo. (**a**) Representative bioluminescence images of individual OV-90 xenograft tumor-bearing NSG mice post CAR T cell injection. (**b**) Quantification of bioluminescence as total flux in photons per second (p/s) of the different experimental groups after CAR T cell injection over 21 d. Data points represent mean ± SEM. Human leucocyte expansion in peripheral blood of injected mice was analyzed by flow cytometry over time. (**c**) Quantification of tumor size of the different experimental groups before and after CAR T cell injection. Data points represent mean ± SEM. (**d**) Human CD45 expressions in blood samples collected at days 7, 14, and 21 post T cell injection were measured. Data are shown as mean ± SEM. (**e**) CAR T cell count among murine CD45 in mouse blood, bone marrow, spleen, and tumor was analyzed, respectively. On day 21 post T cell injection, mice were sacrificed, and the respective organs were collected and dissociated for ex vivo analysis by flow cytometry. Each data point represents an individual mouse, and horizontal lines represent the mean of the respective group.

In order to assess in vivo functionality as well as specificity of the modified FOLR1 CAR A vector, NSG mice were subcutaneously injected with OV-90 wt or OV-90 *FOLR1* KO cells, respectively. Following tumor establishment, anti-FOLR1 CAR T cells were intravenously injected at a single dose (1 × 10^7^ CAR T cells, 85% transduction efficacy, Supplementary Figure S7a), and mice were monitored over a period of 21 d, including weekly blood withdrawals (Figure 3a). The CAR T cells were tolerated, and no changes in body weight were observed in the CAR T cell-treated animals (Supplementary Figure S7b). The FOLR1-directed CAR T cells rapidly reduced OV-90 wt tumor burden (Figure 6a–c and Supplementary Figure S7c), confirming the results from the previous study (Figure 3). On the other hand, the injection of FOLR1-targeting CAR T cells did not prevent the OV-90 *FOLR1* KO tumors from growing out (Figure 6a–c and Supplementary Figure S7c).

At the end of this study, the remaining xenografts were collected and dissociated. Cells were analyzed for FOLR1 expression indicating the presence of target cells and GFP expression to assess the cellular composition of the xenograft (Supplementary Figure S7d). OV-90 wt xenografts treated with CAR T cells contained less FOLR1-expressing tumor cells whereas Mock treated and untreated tumors retained FOLR1 expression. Additionally, CAR T cell-treated OV-90 xenografts were mainly composed of non-GFP-expressing cells, i.e., non-tumor cells. This is in contrast to the OV-90 Mock and the untreated group as well as the OV-90 *FOLR1* KO xenograft groups, which were composed primarily of GFP-expressing tumor cells. This antigen-dependent activity of the CAR T cells could be correlated with the expansion of human CD45- and CAR-expressing cells (Figure 6d,e). These findings suggest that anti-FOLR1 CAR T cells can penetrate into FOLR1-proficient tumors and eradicate FOLR1-expressing tumor cells.

Additionally, bone marrow, spleen, and remaining tumor tissue were collected and dissociated to single-cell suspensions at the end of this study. Subsequently, CAR T cells were detected in blood, spleen, bone marrow, and xenograft tumors via flow cytometry (Figure 6e). Thus, anti-FOLR1 CAR T cells can persist in peripheral blood and migrate to the spleen and bone marrow after antigen encounter. Moreover, anti-FOLR1 CAR T cells and Mock T cells were present in persisting CD8 T cell phenotypes, i.e., naïve T cells, stem cell-like (T_SCM_), and central memory (T_CM_) in blood, spleen, and bone marrow in the absence of FOLR1 expression. Upon antigen encounter, anti-FOLR1 CAR T cells differentiated into effector cells like effector memory (T_EM_) and effector memory cells re-expressing CD45RA (T_EMRA_) and were detected in blood, spleen, and bone marrow. Within the tumors, which were left post T cell infusion, more effector and less memory T cells were detected as expected for anti-FOLR1 CAR T cells in an FOLR1-expressing tumor. In the absence of FOLR1 more anti-FOLR1 CAR T cells remain in a memory phenotype (Supplementary Figure S7e). The presence of effector cells in the mock groups and also the increase in effector cells or reduction in memory T cells, respectively, in the anti-FOLR1 CAR T cell group in FOLR1-deficient tumors may suggest a human TCR-mediated T cell activation against murine antigens as described previously [32].

Analysis of cytokine secretion over 21 d post CAR T cell infusion revealed elevated levels of IFN-gamma and IL-9 at day 7 in the FOLR1 CAR T-treated group bearing OV-90 wt xenografts (Supplementary Figure S7f), which correlated with anti-tumor function.

Taken together, anti-FOLR1 CAR T cells induced rapid tumor eradication, CAR T cell proliferation, and short-term persistence. Moreover, our FOLR1 CAR showed antigen-dependent cytokine release and pronounced tumor infiltration.

## 4. Discussion

OvCa is the most deadly gynecological cancer and a leading cause of cancer death in women, indicating a high clinical need. The lack of screening tests for OvCa and symptoms that are usually attributed to other more common conditions, e.g., abdominal bloating, discomfort in the pelvic area, and/or changes in bowel habits, contribute to frequent late-stage diagnoses. Consequently, the disease is advanced and might have already disseminated into the peritoneal cavity, which increases the probability of relapse. Current treatment regimens are debulking surgery, chemotherapy, angiogenesis inhibition via bevacizumab-targeting VEGF-A, and the use of PARP inhibitors, acting on cells bearing homologous DNA repair pathway deficiencies.

New targeted therapies like PARP inhibitors have shown clinical benefits for patients in recent years. And most recently, a FOLR1-directed ADC, mirvetuximab soravtansine, was approved by the FDA for the treatment of adult patients with FOLR1-expressing, platinum-resistant epithelial OvCa [33]. This highlights a positive benefit-risk profile for FOLR1 as a target. Despite clinically meaningful results of these targeted therapies, their effects depend on continuous administration, indicating the need for more persisting treatment options.

One approach for a targeted, efficient, and persisting treatment option is CAR T cell therapy. Genetically modified CAR T cells have shown unseen clinical responses in many patients suffering from hematological diseases [34]. Several clinical trials are assessing the functionality of CAR T cells in OvCa targeting various tumor-associated antigens [35,36].

So far, two trials have been reported to address the safety and feasibility of FOLR1-specific CAR T cells in OvCa. The FOLR1-directed CAR T cells used in these two clinical studies differ from our described FOLR1-targeting CAR T cells in multiple aspects. To improve on the previously reported studies, we applied innovative CAR design strategies and a modified manufacturing procedure. The study of Kershaw and colleagues employed a first-generation CAR with a murine binder (MOv18), which was safe but not efficacious, and CAR T cells did neither localize to the tumor nor persist over time [16]. Kandalaft and colleagues used a second-generation CAR with another murine binder (MOv19) [37]. It will be interesting to see the results of this study, particularly since the first FOLR1-direct CAR study using a murine scFv sequence (MOv18) lacked efficacy, which correlated with a potential humoral immune reaction, i.e., the detection of human anti-mouse antibody against the scFv component of the CAR [16]. In contrast, we selected human sequences and a humanized scFv sequence to prevent immunogenicity against our CAR.

Besides this hypothesis, other aspects might have contributed to the lack of efficacy and the lack of CAR T cell persistence in these first clinical studies, such as the CAR design, manufacturing, as well as application route. The initial CAR used by Kershaw and colleagues was delivered by a retroviral vector and driven by a viral SV40 promoter. Kandalaft and colleagues use a lentiviral vector and a viral CMV promoter. It is worth noting that viral promoters used for transgene expression were shown to be silenced by extensive methylation [38,39]. This effect may decrease CAR expression over time. In contrast, our lentiviral vector-based CAR is under the control of a physiological human promoter to reduce the risk of CAR downregulation.

Moreover, Kershaw and colleagues used undisclosed leader, spacer, and transmembrane sequences, which can be relevant for proper CAR function [40]. Kandalaft et al. employ human CD8α leader, hinge, and transmembrane sequences, which are similar to our CAR construct. Also, Kershaw et al. employed the gamma chain of the FcR as the activation domain. In our second-generation CAR, we used the CD3zeta domain for activation with the 4-1BB co-stimulatory domain, which was shown to be superior to first-generation CARs [41]. Kandalaft et al. are currently working with a second-generation CAR based on 41BB, similar to our approach, which promotes CD8+ cytotoxic lymphocytes [42] and T cell persistence [43].

Manufacturing of CAR T cells varies also between the previous studies. Kershaw and colleagues used a combination of CD3 and IL-2 to culture CAR T cells. Kandalaft et al. adapted their process to a combination of CD3, CD28, and IL-2 to culture CAR T cells. Providing a co-stimulation signal via CD28 has been shown to deliver essential signals for T cell activation [44]. Although we report activation and stimulation via CD3 and CD28, too, CAR T cells are expanded in IL-7 and IL-15 to promote persistence of central memory or stem cell-like T cell phenotypes [45].

The ongoing clinical trial proposed by Kandalaft et al. (NCT03585764) is a phase I study to establish the safety and feasibility of intraperitoneally administered, lentivirally transduced MOv19-BBz CAR T cells with or without lymphodepletion chemotherapy. The results from this study are awaited and will contribute to our understanding of the safety of CAR T cell application in patients with OvCa.

Here, we report promising preclinical data on a novel FOLR1-directed CAR T cell candidate. Initially, FOLR1 was identified as a surface target for OvCa with high expression levels on tumor cells, homogenous expression within individual tumors, and common expression across multiple patients. Moreover, FOLR1 expression is low or absent in healthy tissues, and expression is apically polarized, which may make FOLR1 inaccessible for drugs in circulation, confirming a beneficial safety profile for FOLR1. Next, we designed a series of FOLR1-targeting CAR constructs. After in vitro and in vivo assessment for specificity and efficacy, we selected a lead CAR candidate. We optimized the lead CAR construct and successfully performed advanced in vitro and in vivo characterization, including ex vivo assays, reflecting the suppressive tumor microenvironment. Further studies are needed to determine whether this potential CAR T cell product is safe and effective in OvCa patients.

## 5. Conclusions

Our findings confirm that FOLR1 is a reasonable and safe target for OvCa. Furthermore, extensive assessment of in vitro, in vivo, and advanced ex vivo models revealed the specificity, efficacy, and persistence of our novel FOLR1-specific CAR. In conclusion, this article provides preclinical data to initiate the clinical translation of our FOLR1-targeting CAR T cell candidate.

## 6. Patents

Patent applications covering aspects of this work are filed.

## Data Availability

All data generated or analyzed during this study are included in this article. Further inquiries can be directed to the corresponding author upon reasonable request.

## Supplementary Materials

The following supporting information can be downloaded at: https://www.mdpi.com/article/10.3390/cancers16020333/s1, Figure S1: Ultrahigh-content imaging revealing FOLR1 expression in high-grade serous epithelial ovarian cancer and healthy tissues; Figure S2: CRISPR/CAS9-mediated FOLR1 knock-out in OV-90 cells is validated at genome, transcript, and protein level; Figure S3: Additional healthy donor-derived FOLR1-directed CAR T cells confirm in vitro co-culture kinetics; Figure S4: Anti-FOLR1 CAR T cells are highly specific for human FOLR1; Figure S5: Anti-FOLR1 CAR T candidates efficiently eradicate OV-90 wt tumors in NSG mice; Figure S6: Differential FOLR1 expression in ovarian cancer cell lines; Figure S7: Improved anti-FOLR1 CAR T cells specifically eradicate OV-90 FOLR1-expressing tumors in NSG mice; Table S1: Reagent list.
