# Supplementary material for: Preclinical Evaluation of Novel Folate Receptor 1-Directed CAR T Cells for Ovarian Cancer
Source: Cancers (Basel). 2024 Jan 12;16(2):333. doi: 10.3390/cancers16020333 (PMC10813853; doi:10.3390/cancers16020333)
Supplement: Supplementary file 1 [file cancers-16-00333-s001.zip › cancers-2788386-supplementary.pdf]

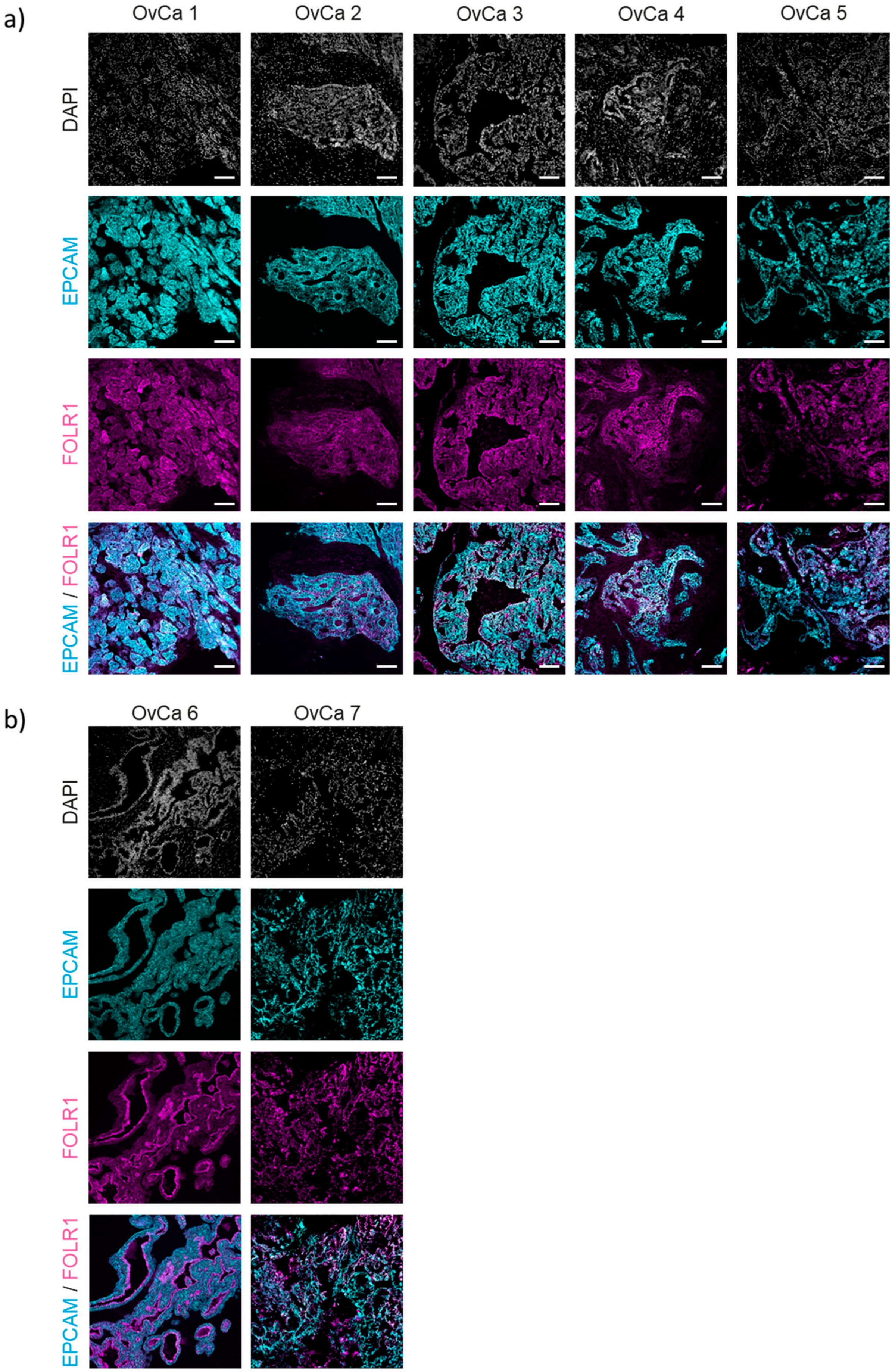

a)
OvCa 1
OvCa 2
OvCa 3
OvCa 4
OvCa 5
DAPI
EPCAM
FOLR1
EPCAM / FOLR1
b)
OvCa 6
OvCa 7
DAPI
EPCAM
FOLR1
EPCAM / FOLR1

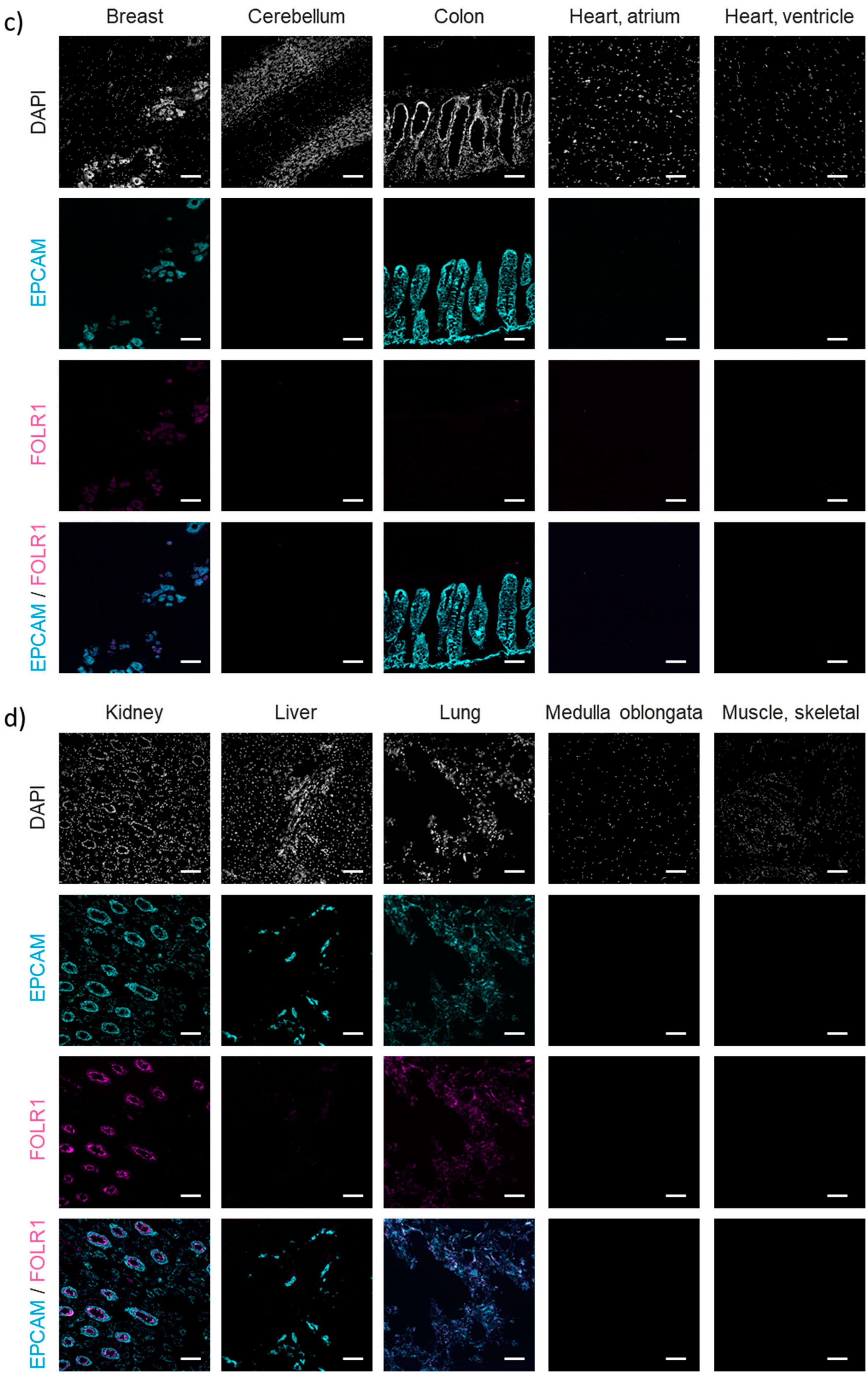
c)
Breast
Cerebellum
Colon
Heart, atrium
Heart, ventricle
DAPI
EPCAM
FOLR1
EPCAM / FOLR1
d)
Kidney
Liver
Lung
Medulla oblongata
Muscle, skeletal
DAPI
EPCAM
FOLR1
EPCAM / FOLR1

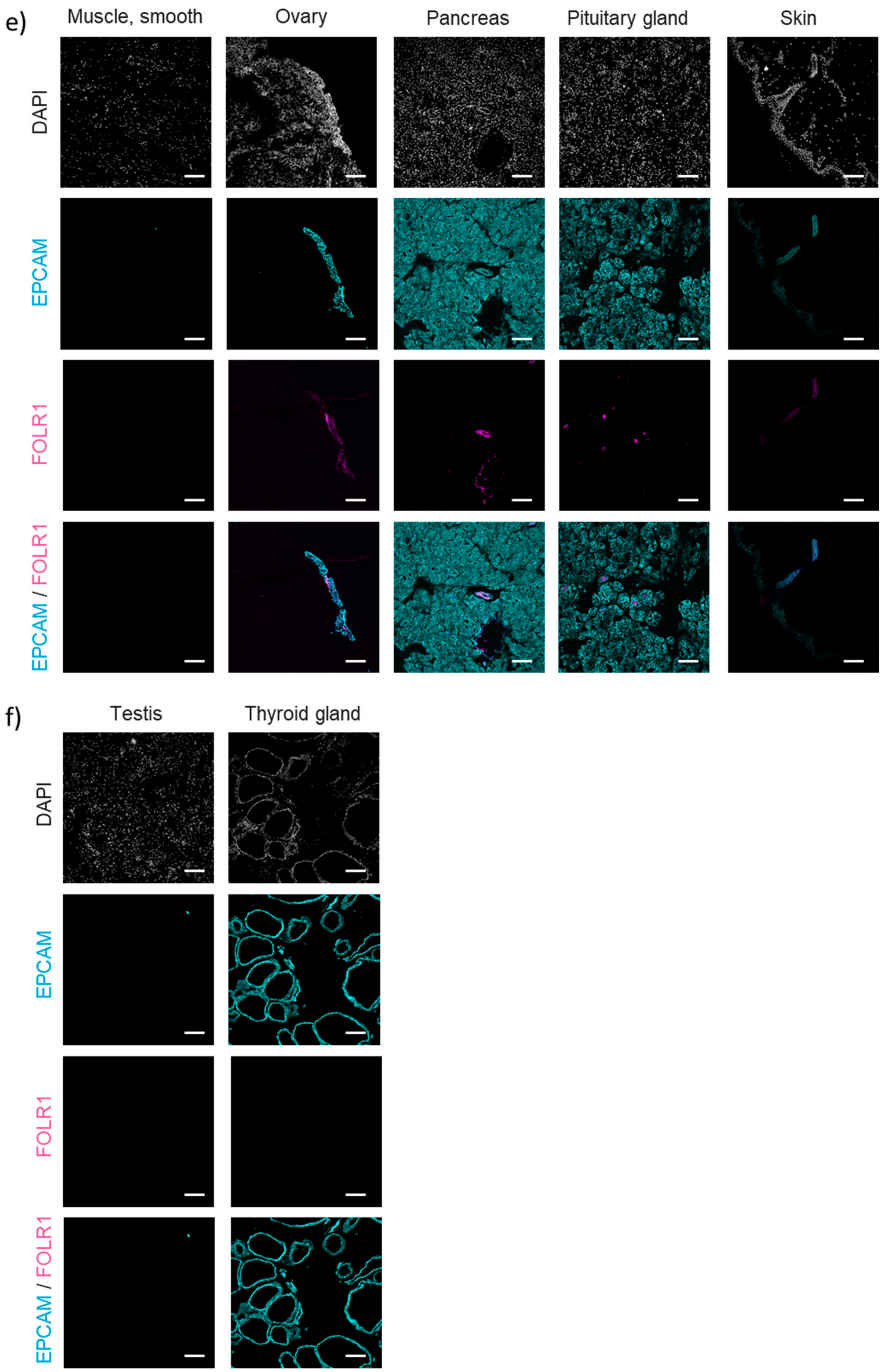
e)
Muscle, smooth
Ovary
Pancreas
Pituitary gland
Skin
DAPI
EPCAM
FOLR1
EPCAM / FOLR1
f)
Testis
Thyroid gland
DAPI
EPCAM
FOLR1
EPCAM / FOLR1

Supplementary Figure S1. Ultrahigh-content imaging revealing FOLR1 expression in high-grade serous epithelial ovarian cancer and healthy tissues.

(**a, b),** Fresh-frozen HGSOC samples (each number indicates an individual patient sample) were sliced and analyzed by MICS. Sequential staining of DAPI (white), EPCAM (cyan), and FOLR1 (magenta) are represented, as well as co-expression of EPCAM and FOLR1 in the lower panels. Healthy tissues samples (**c**) breast, cerebellum, colon, heart-atrium, heart-ventricle, (**d**) kidney, liver, lung, medulla oblongata, skeletal muscle, (**e**) smooth muscle, ovary, pancreas, pituitary gland, skin, (**f**) testis and thyroid gland were sliced and analyzed by MICS. Sequential staining of DAPI (white), EPCAM (cyan), and FOLR1 (magenta) are represented, as well as co-expression of EPCAM and FOLR1 in the lower panels. Scale bar represents 100 µm.

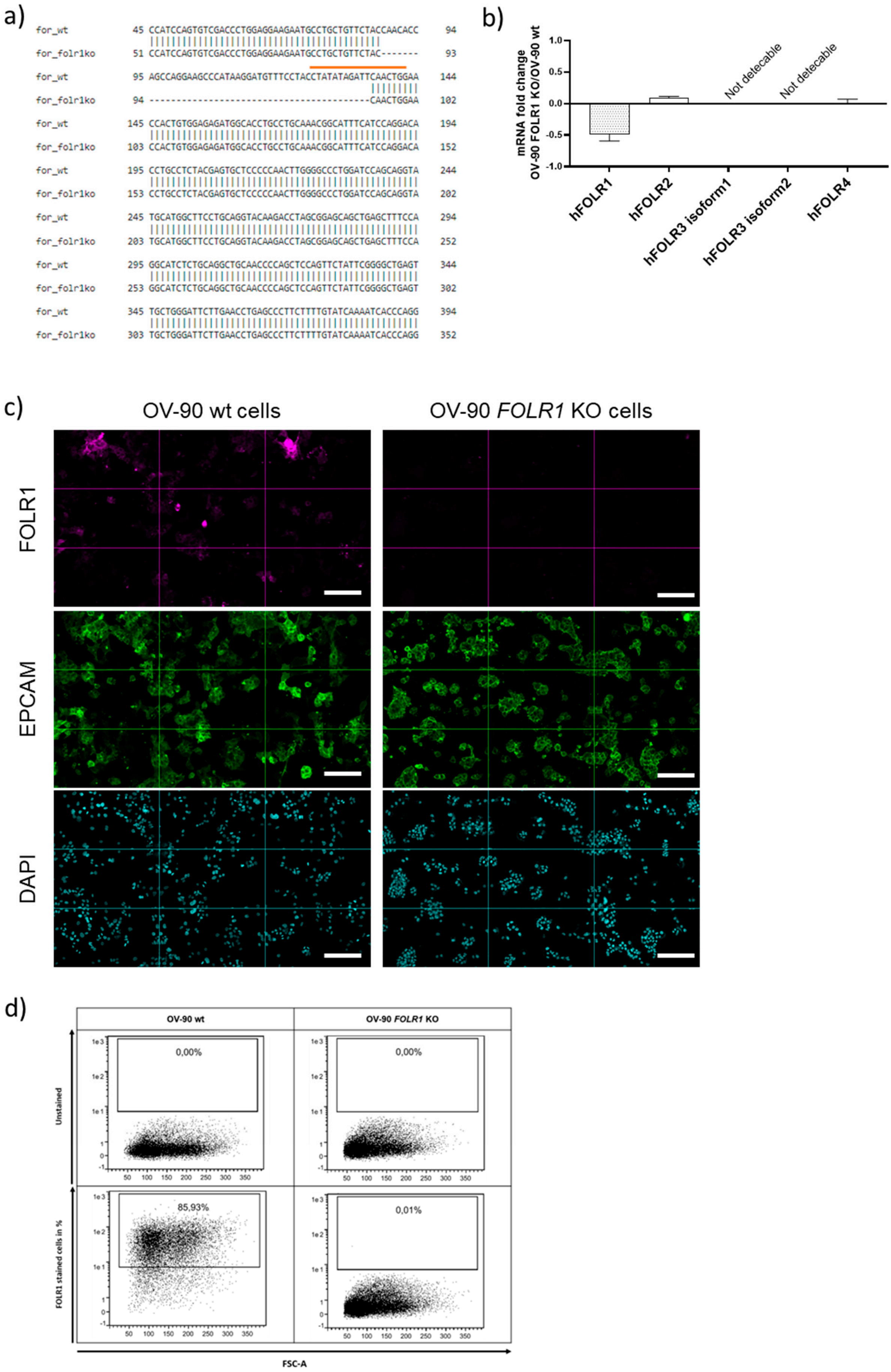

a)
for_wt 45 CCATCCAGTGTCGACCCTGGAGGAAGAATGCCTGCTGTTCTACCAACACC 94
for_folr1ko 51 CCATCCAGTGTCGACCCTGGAGGAAGAATGCCTGCTGTTCTAC------- 93
for_wt 95 AGCCAGGAAGCCCATAAGGATGTTTCCTACCTATATAGATTCAACTGGAA 144
for_folr1ko 94 ---CAACTGGAA 102
for_wt 145 CCACTGTGGAGAGATGGCACCTGCCTGCAAACGGCATTTCATCCAGGACA 194
for_folr1ko 103 CCACTGTGGAGAGATGGCACCTGCCTGCAAACGGCATTTCATCCAGGACA 152
for_wt 195 CCTGCCTCTACGAGTGCTCCCCCAACTTGGGGCCCTGGATCCAGCAGGTA 244
for_folr1ko 153 CCTGCCTCTACGAGTGCTCCCCCAACTTGGGGCCCTGGATCCAGCAGGTA 202
for_wt 245 TGCATGGCTTCCTGCAGGTACAAGACCTAGCGGAGCAGCTGAGCTTTCCA 294
for_folr1ko 203 TGCATGGCTTCCTGCAGGTACAAGACCTAGCGGAGCAGCTGAGCTTTCCA 252
for_wt 295 GGCATCTCTGCAGGCTGCAACCCCAGCTCCAGTTCTATTCGGGGCTGAGT 344
for_folr1ko 253 GGCATCTCTGCAGGCTGCAACCCCAGCTCCAGTTCTATTCGGGGCTGAGT 302
for_wt 345 TGCTGGGATTCTTGAACCTGAGCCCTTCTTTTGTATCAAAATCACCCAGG 394
for_folr1ko 303 TGCTGGGATTCTTGAACCTGAGCCCTTCTTTTGTATCAAAATCACCCAGG 352
b)
mRNA fold change
OV-90 FOLR1 KO/OV-90 wt
1.0
0.5
0.0
-0.5
-1.0
Not detecable
Not detecable
hFOLR1
hFOLR2
hFOLR3 isoform1
hFOLR3 isoform2
hFOLR4
c)
OV-90 wt cells
OV-90 FOLR1 KO cells
FOLR1
EPCAM
DAPI
d)
OV-90 wt
OV-90 FOLR1 KO
Unstained
FOLR1 stained cells in %
0,00%
0,00%
85,93%
0,01%
FSC-A

Supplementary Figure S2. CRISPR/CAS9-mediated *FOLR1* knock-out in OV-90 cells is validated at genome, transcript, and protein level. Deletion in *FOLR1* led to homozygous knock-out in OV-90 cells. (**a**) Alignment of OV-90 wt (for_wt) and OV-90 *FOLR1* KO (for_folrko) genomic DNA sequences using EMBOSS needle algorithm. The sequence highlighted with orange line corresponds to the guide RNA sequence used for the CRISPR/CAS9-mediated knock-out of FOLR1. (**b**) Relative mRNA expression of folate receptor variants in OV-90 *FOLR1* KO compared to OV-90 wt cells. FOLR transcripts were amplified with primer pairs for different folate receptors variants. Individual gene expression is analyzed by ΔΔCt method, normalized to GAPDH mRNA expression and fold change is relative to OV-90 wt mRNA levels. Data is shown as mean ± SEM. (**c**) Immune fluorescence anti-FOLR1 staining of OV-90 wt as well as OV-90 *FOLR1* KO cells. Cells were analyzed for the expression of FOLR1 and EPCAM. DAPI visualizes nuclei. Scale bar represents 100 µm. (**d**) Flow cytometric analysis of FOLR1 expression in OV-90 wt as well as OV-90 *FOLR1* KO cells.

a)

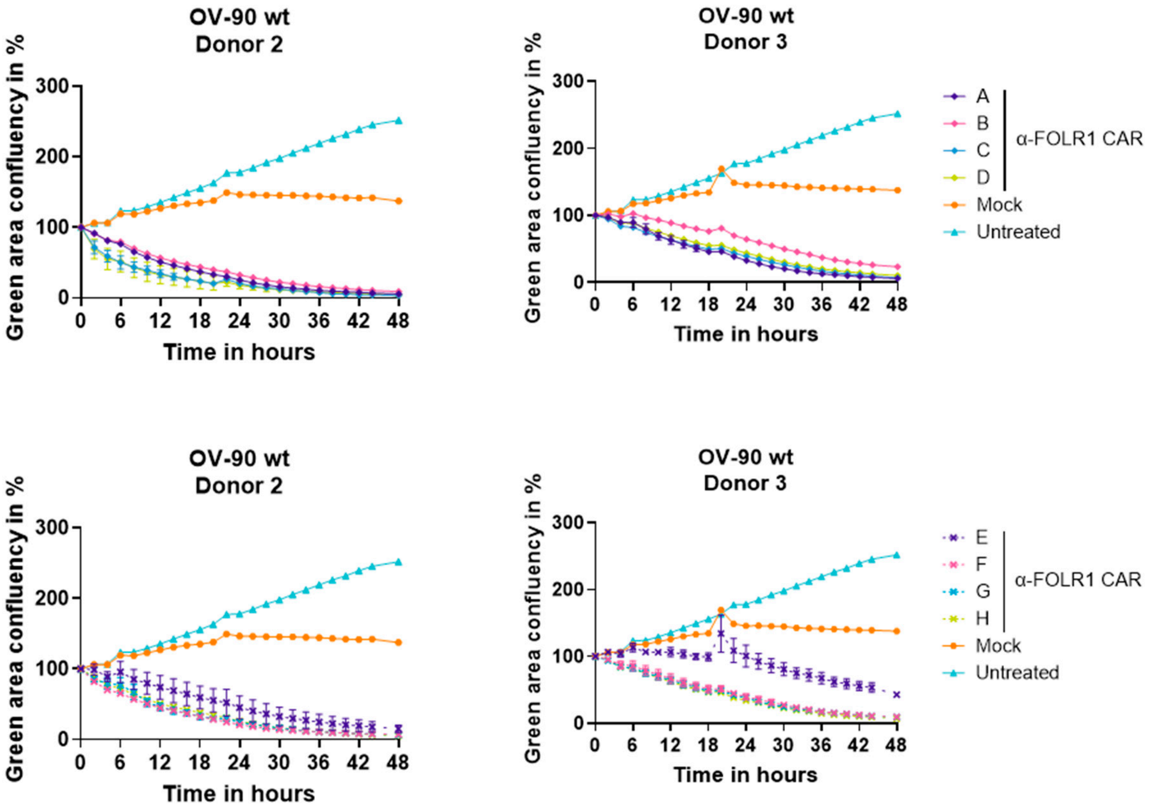
OV-90 wt
Donor 2
OV-90 wt
Donor 3
Green area confluency in %
Time in hours
0
6
12
18
24
30
36
42
48
100
200
300
A
B
C
D
α-FOLR1 CAR
Mock
Untreated
E
F
G
H

b)

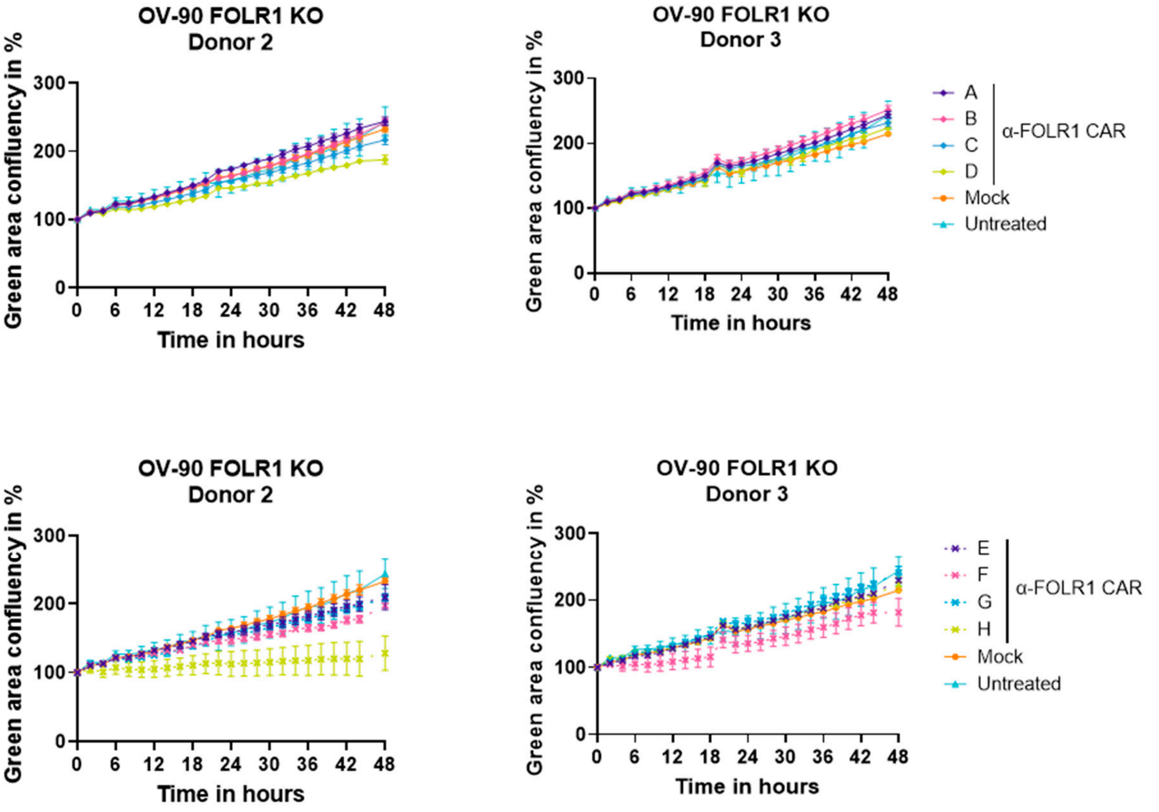
OV-90 FOLR1 KO
Donor 2
OV-90 FOLR1 KO
Donor 3
Green area confluency in %
Time in hours
0
6
12
18
24
30
36
42
48
100
200
300
A
B
C
D
α-FOLR1 CAR
Mock
Untreated
E
F
G
H

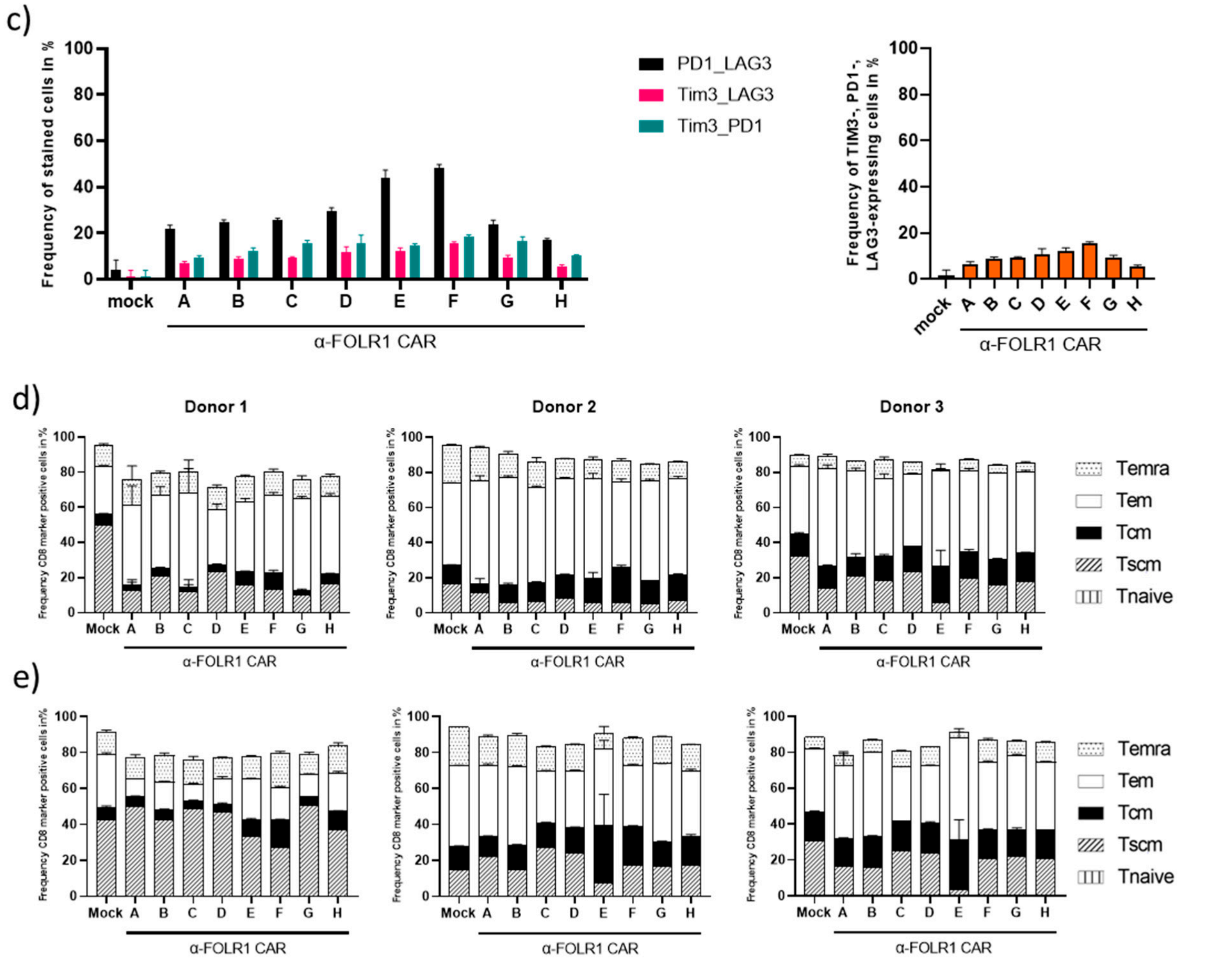

Supplementary Figure S3. Additional healthy donor-derived FOLR1-directed CAR T cells confirm *in vitro* co-culture kinetics. (**a**) Representative killing kinetic of OV-90 wt in co-culture with the different FOLR1-specific CAR T cells at an effector to target ratios of 2:1. Each datapoint represents mean +/- SEM (n=3). (**b**) Representative killing kinetic of OV-90 *FOLR1* KO in co-culture with the different FOLR1-specific CAR T cells at an effector to target ratios of 2:1. Each datapoint represents mean +/- SEM (n=3). (**c**) Anti-FOLR1 CAR T cells (CD8 T cells) were phenotyped after co-cultivation. Anti-FOLR1 CAR T cells or untransduced T cells (Mock) from three independent donors were co-cultured with (**d**) OV-90 wt cells or (**e**) OV-90 FOLR1 KO cells at an effector cell : target cell ratio of 2:1. After 48 hours of co-culture, T cells were assessed for phenotype markers expression by flow cytometry. T cell subtypes were defined as following: Tscm – CD45RO- CD197+ CD62L+ CD95+, Tnaive - CD45RO- CD197+ CD62L+ CD95-, Temra – CD45RO- CD197- CD62L- CD95+, Tem – CD45RO+ CD62L- CD95+, and Tcm – CD45RO+ CD62L+ CD95+. Each bar represents mean +/- SEM (n=3).

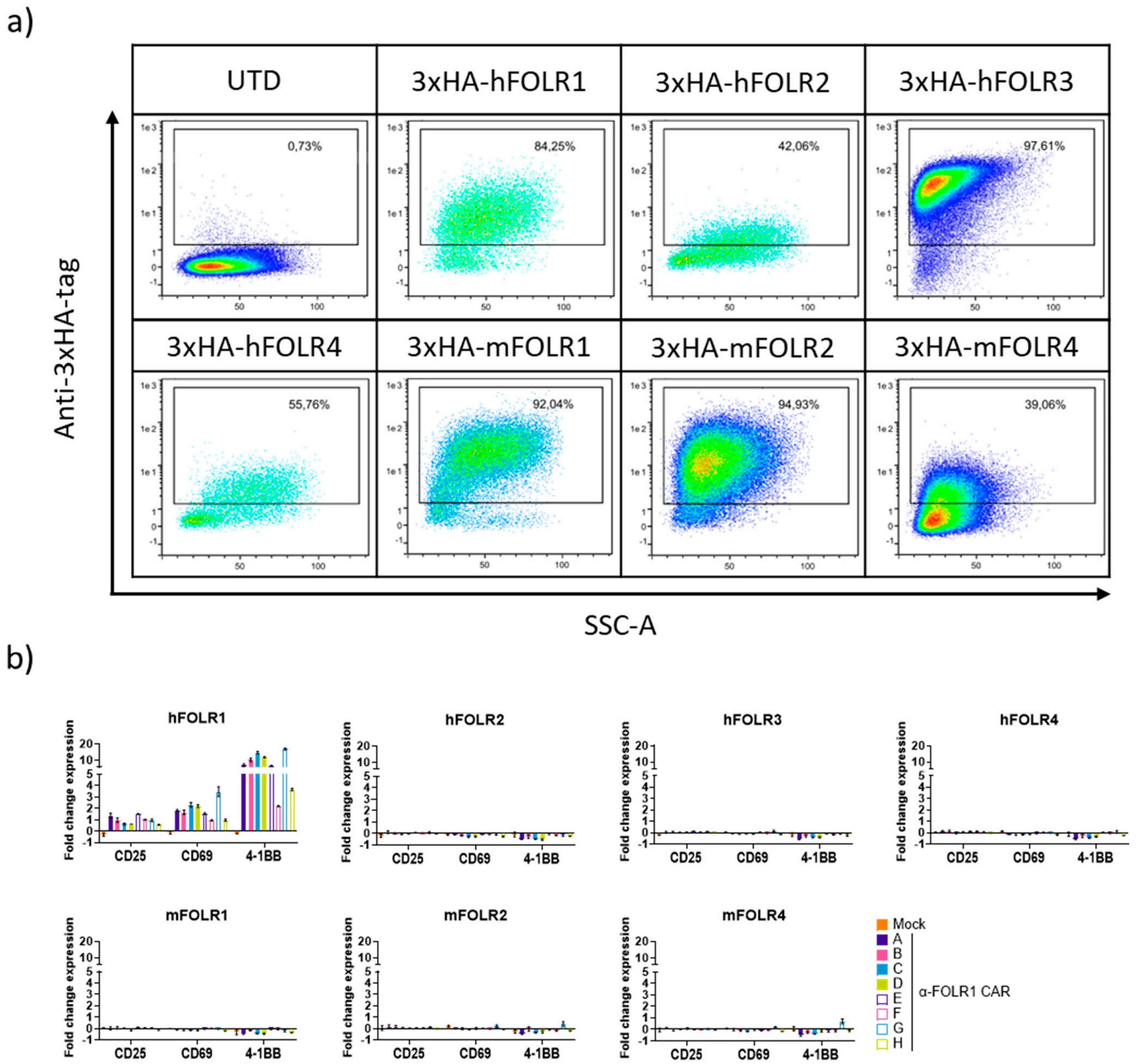

Supplementary Figure S4. Anti-FOLR1 CAR T cells are highly specific for human FOLR1. (**a**) Density plots from flow cytometric analysis of wt Jurkat cells or Jurkat cells expressing different human (h) or mouse (m) FOLR variants. HA-tagged FOLR variants expression was detected via an anti-3xHA-tag staining. (**b**) Activation marker CD25, CD69, and 4-1BB expressed by anti-FOLR1 CAR T cells after 48 hours of co-culture with Jurkat cells expressing different human (h) or mouse (m) FOLR variants, at an effector cell to target cell ratio of 2:1. Data points represent mean of technical triplicates and are presented as fold change relative to the respective T cell co-culture with FOLR-deficient Jurkat wt cells. Each bar represents the mean +/- SEM (n=3).

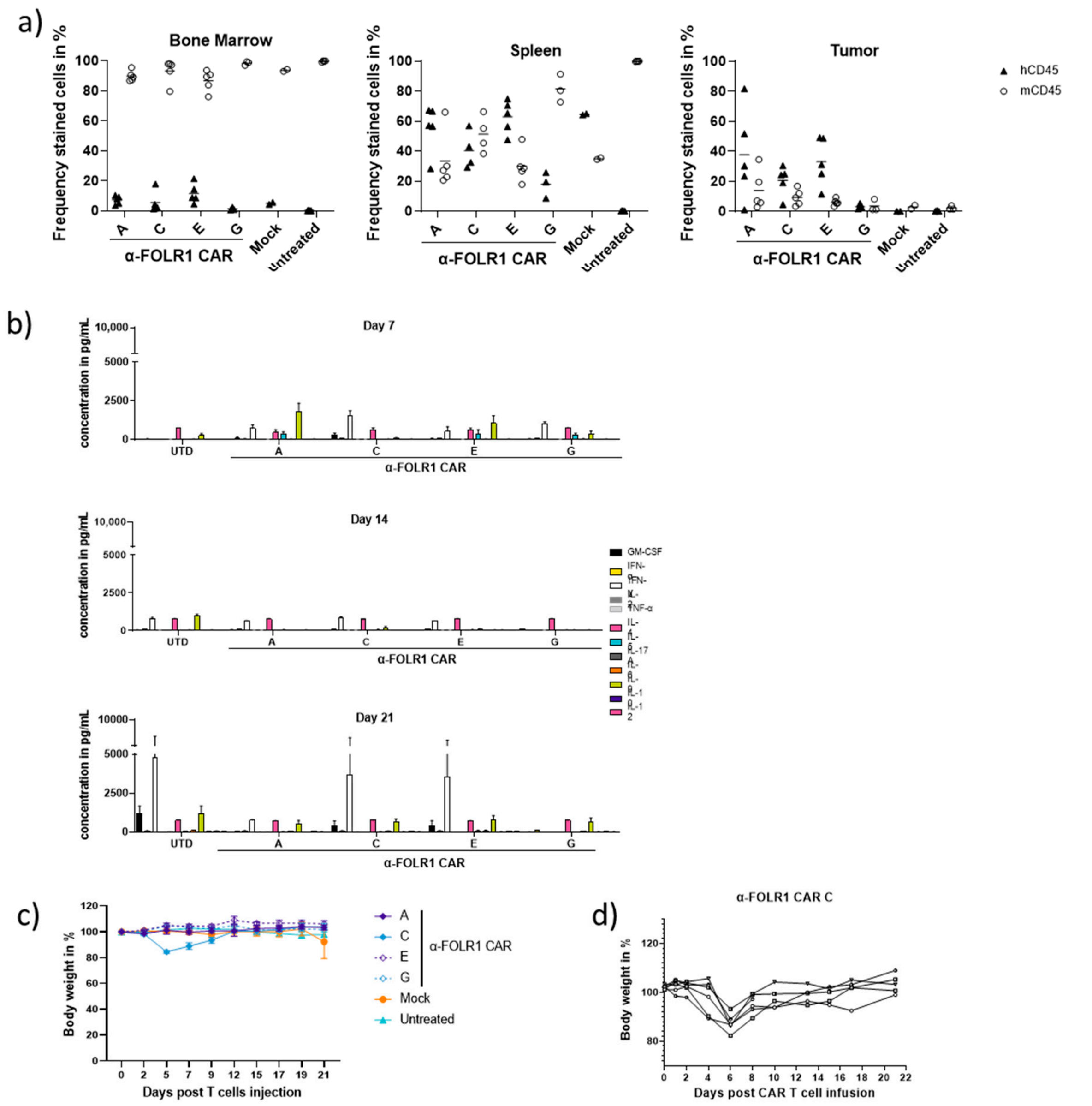

Supplementary Figure S5. Anti-FOLR1 CAR T candidates efficiently eradicate OV-90 wt tumors in NSG mice. (**a**) Mouse and human leucocytes composition in mouse bone marrow, spleen, and tumor was analyzed. On day 21 post T cell injection mice were sacrificed and the respective organs collected and dissociated. Single cell suspensions of the organs were analyzed *ex vivo* by flow cytometry for murine or human CD45 expression, respectively. Each data point represents an individual mouse and horizontal lines represent the mean of the respective group. (**b**) Secretion of human cytokines was analyzed in peripheral blood samples over time. Plasma was isolated from blood samples collected at day 7, 14, and 21 post T cell injection and cytokine levels were subsequently determined with the human MACSplex Cytokine 12 Kit. Data is shown as mean ± SEM. (**c**) Body weight of tumor-bearing mice was monitored over 21 days post CAR T cell injection. Each line corresponds to an individual mouse. (**d**) Tumor-free NSG mice were intravenously injected with $1x10^7$anti-FOLR1 CAR T cells (α-FOLR1 CAR C) produced in CliniMACS Prodigy® to assess tolerability. Weight was monitored over the 21 days post injection. Each line corresponds to a single mouse.

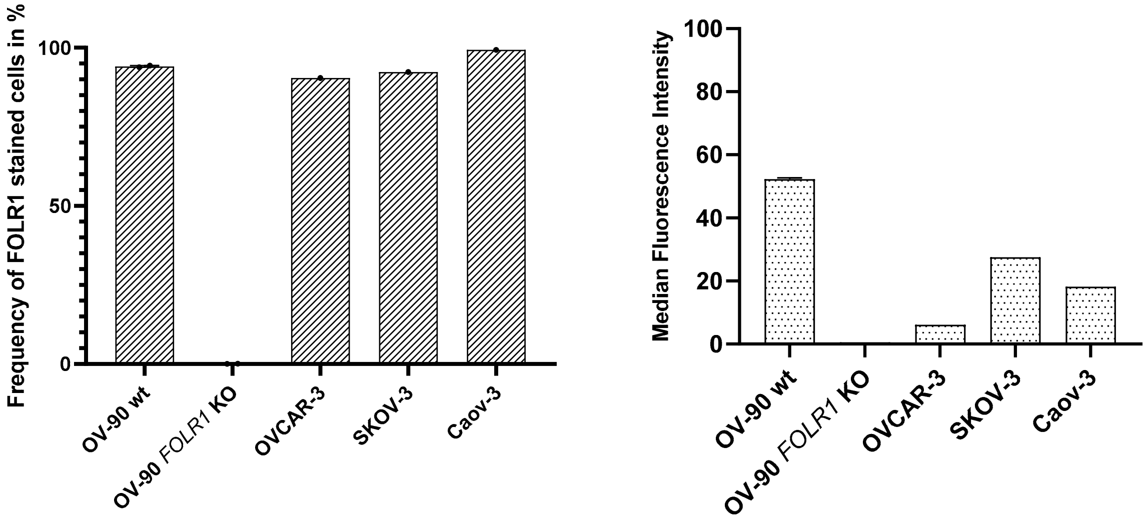

Supplementary Figure S6. Differential FOLR1 expression in ovarian cancer cell lines. Various ovarian cancer cell lines were analyzed for FOLR1 expression via flow cytometry.

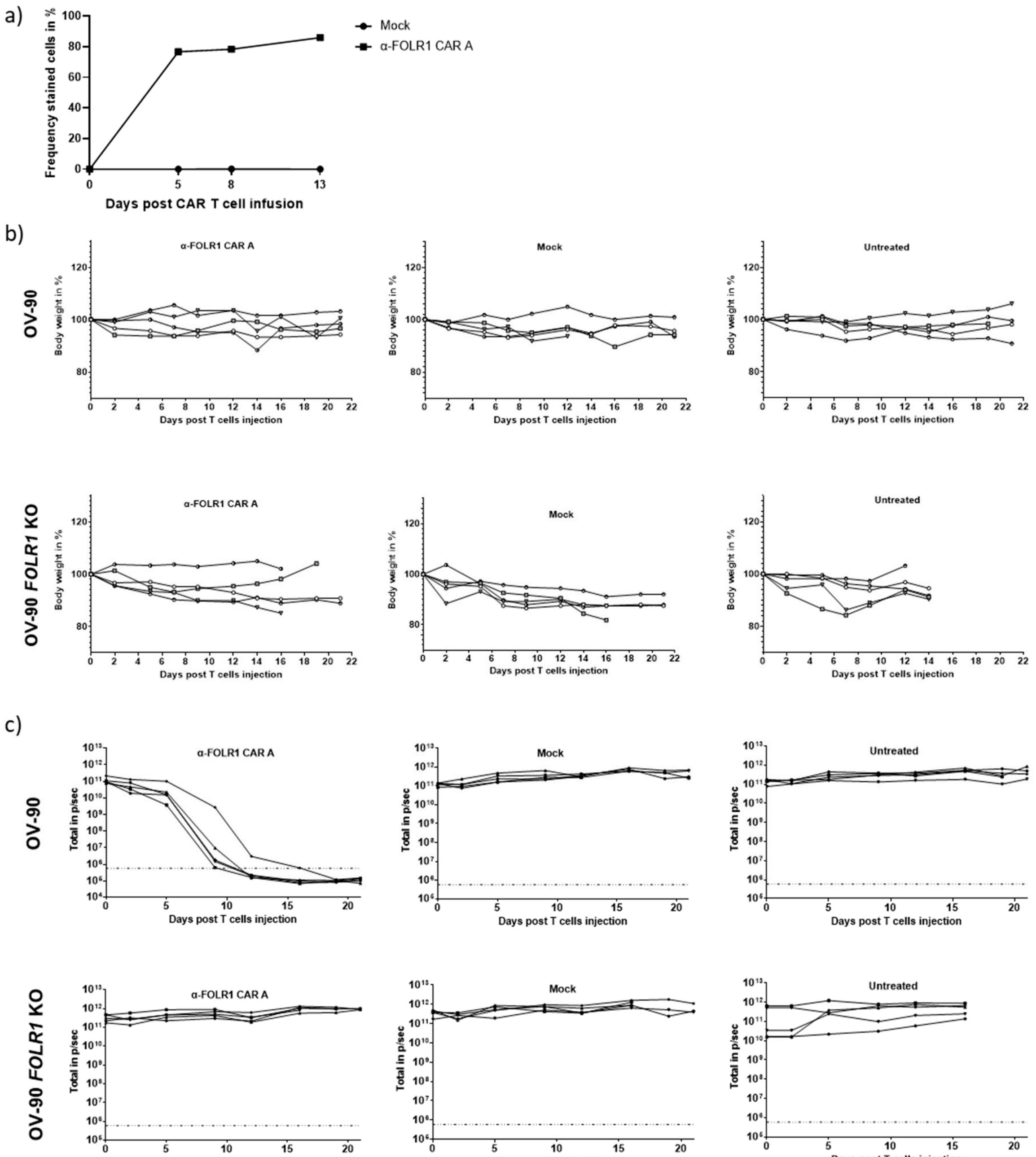

a)
Frequency stained cells in %
Days post CAR T cell infusion
Mock
α-FOLR1 CAR A
b)
OV-90
OV-90 FOLR1 KO
α-FOLR1 CAR A
Mock
Untreated
Body weight in %
Days post T cells injection
c)
OV-90
OV-90 FOLR1 KO
α-FOLR1 CAR A
Mock
Untreated
Total in p/sec
Days post T cells injection

d)

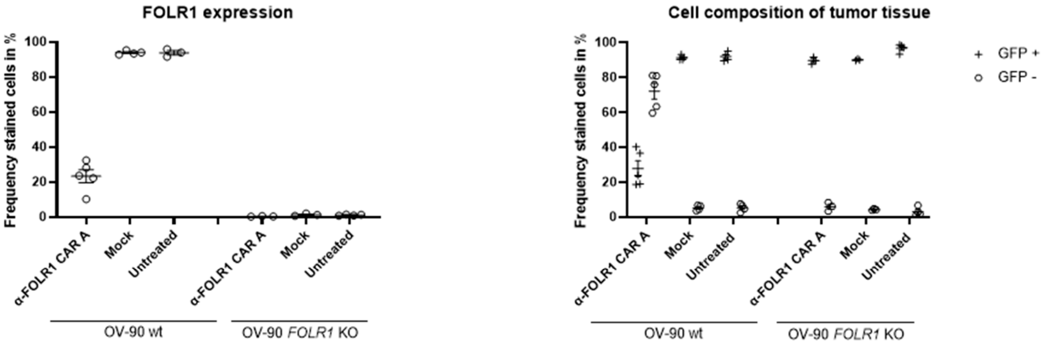
FOLR1 expression
Frequency stained cells in %
α-FOLR1 CAR A
Mock
Untreated
OV-90 wt
OV-90 *FOLR1* KO
Cell composition of tumor tissue
GFP +
GFP -

e)

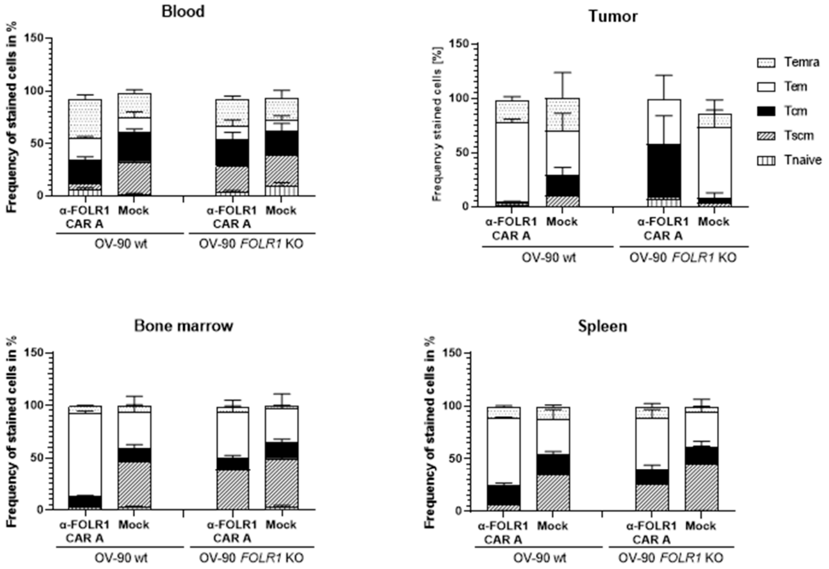
Blood
Tumor
Bone marrow
Spleen
Frequency of stained cells in %
Frequency stained cells [%]
α-FOLR1 CAR A
Mock
OV-90 wt
OV-90 *FOLR1* KO
Temra
Tem
Tcm
Tscm
Tnaive

f)

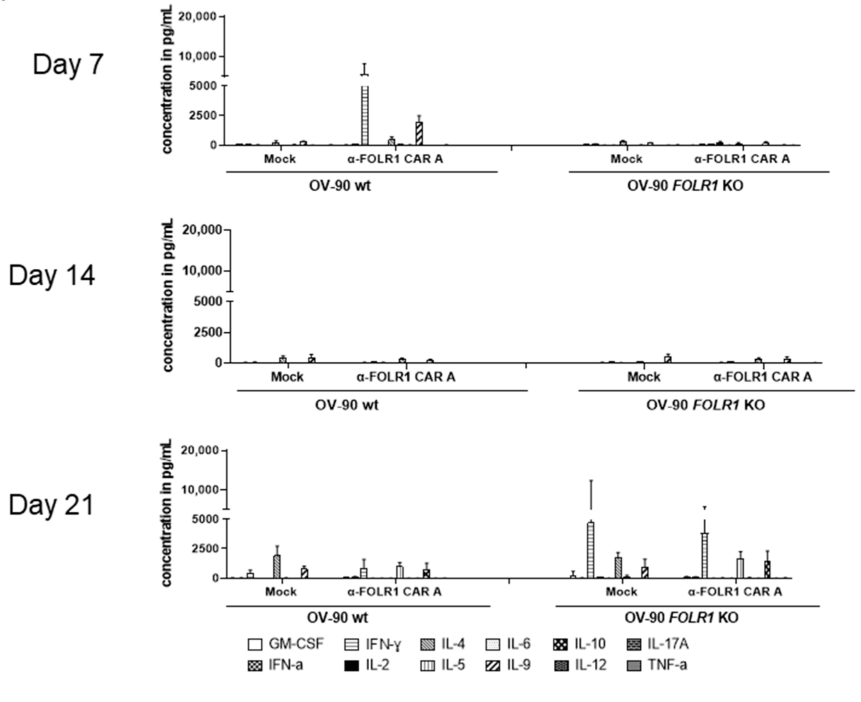
Day 7
Day 14
Day 21
concentration in pg/mL
Mock
α-FOLR1 CAR A
OV-90 wt
OV-90 *FOLR1* KO
GM-CSF
IFN-γ
IL-4
IL-6
IL-10
IL-17A
IFN-a
IL-2
IL-5
IL-9
IL-12
TNF-a

Supplementary Figure S7. Improved Anti-FOLR1 CAR T cells specifically eradicate OV-90 FOLR1 expressing tumors in NSG mice.

NSG mice were injected with OV-90 wt or OV-90 *FOLR1* KO cells subcutaneously and after 21 days intravenously injected with anti-FOLR1 CAR T cells ($1x10^7$ total T cells; Anti-FOLR1 CAR A) or untransduced T cells (Mock) produced in CliniMACS Prodigy® to assess anti-tumor function. (**a**) Frequency of CAR expressing T cells was monitored over time by flow cytometry (FOLR1-Fc fusion protein staining). (**b**) Body weight of mice bearing OV-90 wt or OV-90 *FOLR1* KO xenograft tumors, respectively, was monitored over 21 days post CAR T cell injection. Each line corresponds to an individual mouse. (**c**) Quantification of bioluminescence as total flux in photons per seconds (p/s) of individual mice bearing OV-90 wt or OV-90 *FOLR1* KO xenograft tumors, respectively, after CAR T cell injection over 21 days. Each line represents an individual mouse. (**d**) FOLR1 expression and cell composition in remaining tumor tissues at study endpoint was assessed. Composition of the xenograft tissue represented as GFP positive tumor cells and GFP negative non-tumor cells is depicted in the right panel. *Ex vivo* analysis were performed at the study endpoint, 21 days after CAR T cell injection for all groups. (**e**) Secretion of human cytokines was analyzed in peripheral blood samples over time. Plasma was isolated from blood samples collected at day 7, 14, and 21 post T cell injection and cytokine levels were subsequently determined with the human MACSplex Cytokine 12 Kit. Data is shown as mean ± SEM of values. (**f**) Human CD8 T cells from mouse blood, bone marrow, spleen, and tumor were phenotyped. On day 21 post T cell injection mice were sacrificed and the respective organs collected and dissociated. Single cell suspensions of the organs were analyzed *ex vivo*. T cells were assessed for phenotype marker expression by flow cytometry. T cell subtypes were defined as following: Tscm: CD45RO-, CD197+, CD62L+, CD95+; Tnaive: CD8+ or CD4+, CD45RO-, CD197+, CD62L+, CD95-; Temra: CD45RO-, CD197-, CD62L-, CD95+; Tem: CD45RO+, CD62L-, CD95+; and Tcm: CD45RO+, CD62L+, CD95+. Each bar represents mean +/- SEM of the respective group.

Supplementary Table S1 Reagent list

**Catalog no.**	**product name**	**provider**
170-076-631	20 mL Reagent Bag	Miltenyi Biotec
68982-0643-02	25% HSA	octapharma
130-111-568	7-AAD Staining Solution	Miltenyi Biotec
1072534	Alt-R® CRISPR-Cas9 tracrRNA, 100 nmol	Integrated DNA Technologies
1081058	Alt-R™ S.p. Cas9 Nuclease V3, 100 µg	Integrated DNA Technologies
130-092-820	Annexin V Binding Buffer	Miltenyi Biotec
130-093-060	Annexin V conjugates	Miltenyi Biotec
153304	Anti-Folr2 (Folate receptor 2) Antibody, anti-mouse, 10/FR2	Biolegend

391704	Anti-Folr2 (Folate receptor 2) Antibody, anti-human, clone 94b/FOLR2	Biolegend
04-968	Anti-Folr4 (Folate receptor 4) Antibody, anti-human, clone 5E12.2	Sigma-Aldrich
130-110-952	Biotin Antibody, REAfinity™ (REA746) - APC	Miltenyi Biotec
130-110-951	Biotin Antibody, REAfinity™ (REA746) - PE	Miltenyi Biotec
FO1-H82F9	Biotinylated Human FOLR1 Protein, Fc,Avitag™ (MALS verified)	Acro Biosystems
B6542-5MG	Brefeldin A aus Penicillium brefeldianum	Sigma-Aldrich
HTB-75	Caov-3 (HTB-75)	ATCC
130-123-553	HA Antibody - APC	Miltenyi Biotec
130-110-764	CD137 Antibody, anti-human, REAfinity™ (REA765) - APC	Miltenyi Biotec
130-118-549	CD223 Antibody, anti-human, REAfinity™ (REA351) - Vioblue	Miltenyi Biotec
130-116-205	CD25 Antibody, anti-human, REAfinity™ (REA945) - PEVio770	Miltenyi Biotec
130-120-385	CD279 (PD1) Antibody, anti-human, PE-Vio® 770, REAfinity™ - PEVio770	Miltenyi Biotec
130-113-138	CD3 Antibody, anti-human, REAfinity™ (REA613) - FITC	Miltenyi Biotec
130-110-999	CD326 (EpCAM) Antibody, anti-human, REAfinity™ (REA764) - PE	Miltenyi Biotec
130-111-004	CD326 (EpCAM) Antibody, anti-human, REAfinity™ (REA764) - Vioblue	Miltenyi Biotec
130-119-781	CD366 (TIM-3) Antibody, anti-human, REAfinity™ (REA635) - APC	Miltenyi Biotec
130-113-230	CD4 Antibody, anti-human, REAfinity™ (REA623) - Viogreen	Miltenyi Biotec
130-110-637	CD45 Antibody, anti-human, REAfinity™ (REA747) - Vioblue	Miltenyi Biotec
130-110-799	CD45 Antibody, anti-mouse, PE-Vio® 770, REAfinity™ - PEVio770	Miltenyi Biotec
130-120-033	CD45RA Antibody, anti-human, REAfinity™ (REA562) - Vioblue	Miltenyi Biotec

130-113-556	CD45RO Antibody, anti-human, REAfinity™ (REA611) - APC	Miltenyi Biotec
130-113-621	CD62L Antibody, anti-human - PEVio770	Miltenyi Biotec
130-112-610	CD69 Antibody, anti-human, REAfinity™ (REA824) - Vioblue	Miltenyi Biotec
130-110-819	CD8 Antibody, anti-human, REAfinity™ (REA734) - PEVio770	Miltenyi Biotec
200-073-613	CliniMACS Prodigy® Tubing Set 520	Miltenyi Biotec
170-076-614	CliniMACS Prodigy® Tubing Set 620	Miltenyi Biotec
170-076-625	CliniMACS® Electroporation Buffer	Miltenyi Biotec
170-076-327	CliniMACS® Formulation Solution	Miltenyi Biotec
200-070-025	CliniMACS® PBS/EDTA Buffer	Miltenyi Biotec
354277	Corning® Matrigel® hESC-Qualified Matrix, LDEV-free	Corning
130-111-570	DAPI Staining Solution	Miltenyi Biotec
LUCK-100	D-Luciferin, Potassium Salt (Proven and Published®)	GoldBio
L0103	DMEM High Glucose w/ Stable Glutamine w/ Sodium Pyruvate	biowest
69504	DNeasy Blood & Tissue Kit (50)	QIAGEN
BS-2022-500	FCS	Catus Biotech GmbH
ACC 635	Human embryonic kidney 293T cells (HEK293T)	DSMZ
130-095-764	Human IL-15	Miltenyi Biotec
130-095-362	Human IL-7	Miltenyi Biotec
515-HI	Human Male AB Serum	Access Cell Culture
BE17-605E	L-Glutamin	Lonza
130-042-401	LS Columns	Miltenyi Biotec
200-073-903	Luer/Spike Interconnector	Miltenyi Biotec

130-091-376	MACS BSA Stock Solution	Miltenyi Biotec
130-126-719	MACS® Clearing Kit	Miltenyi Biotec
130-128-157	MACS® COPYcheck Kit, human	Miltenyi Biotec
130-106-197	MACSPlex Custom Cytokine Assays	Miltenyi Biotec
130-099-169	MACSPlex Cytokine Kits	Miltenyi Biotec
130-125-800	MACSPlex Cytotoxic T/NK Cell Kit, human	Miltenyi Biotec
130-093-607	MACSQuant® Calibration Beads	Miltenyi Biotec
130-126-865	MACSwell™ Deepwell Plates	Miltenyi Biotec
117-500	MCDB 105 Medium (500 ml)	Sigma-Aldrich
31150030	Medium 199, Earle's Salze	Thermo Fisher
130-097-095	Naive Pan T Cell Isolation Kit, human	Miltenyi Biotec
130-097-096	Naive Pan T Cell Isolation Kit, human	Miltenyi Biotec
303410	Natriumbutyrat	Sigma-Aldrich
HTB-161	NIH:OVCAR-3 (HTB-161)	ATCC
CRL-11732	OV-90 (CRL-11732)	ATCC
130-096-535	Pan T Cell Isolation Kit, human	Miltenyi Biotec
P04-60500	Pancoll human, Density: 1.077 g/ml	PAN-Biotech GmbH
908304	PE anti-FOLR1 (Folate Binding Protein) Antibody	BioLegend
BLD-391704	PE anti-human Folate Receptor beta (FR-beta), Clone: [94b/FOLR2], Mouse, Monoclonal	Biozol
ant-pm-2	Primocin®	InvivoGen
M0494S	Q5 Hot Start High-Fidelity 2X Master Mix	New England BioLabs
28706	QIAquick Gel Extraction Kit (NucleoSpin Gel and PCR Clean-Up Kit)	Qiagen
ab221543	Recombinant Anti-Folate Binding Protein/FBP antibody [EPR20277] (ab221543)	abcam

130-094-183	Red Blood Cell Lysis Solution (10×)	Miltenyi Biotec
L0501	RPMI 1640 w/o L-Glutamine	biowest
HTB-77	SKOV-3 (HTB-77)	ATCC
130-111-160	T Cell TransAct™, human	Miltenyi Biotec
4444556	TaqMan™ Schneller Advanced Master-Mix	ThermoFisher
170-076-306	TexMACS™ GMP Medium	Miltenyi Biotec
14020108926	Tissue Freezing Medium	Leica Biosystems
T4049	Trypsin-EDTA-Lösung	Sigma-Aldrich
